# Transition Metal‐Driven Selectivity in Direct C−H Arylation of Imidazo[2,1‐*b*]Thiazole

**DOI:** 10.1002/open.202400180

**Published:** 2024-07-25

**Authors:** Antonio Del Vecchio, Elisabetta Rosadoni, Lorenzo Ballerini, Angela Cuzzola, Filippo Lipparini, Paolo Ronchi, Sara Guariento, Matteo Biagetti, Marco Lessi, Fabio Bellina

**Affiliations:** ^1^ Dipartimento di Chimica e Chimica Industriale Università di Pisa Via Giuseppe Moruzzi, 13 56124 Pisa Italy; ^2^ Chemistry Research and Drug Design Chiesi Farmaceutici S.p.A 43122 Parma Italy

**Keywords:** imidazo[2,1-b]thiazoles, direct C−H arylation, selectivity, palladium catalysis, copper

## Abstract

A selective direct arylation of the different Csp2‐H bonds of imidazo[2,1‐*b*]thiazole with (hetero) aryl halides can be achieved simply by switching from a palladium catalyst system to the use of stoichiometric amounts of copper. The observed selectivity, also rationalized by DFT calculations, can be explained by a change in the mechanistic pathways between electrophilic palladation and base‐promoted C−H metalation.

## Graphical abstract

The present work reports on the direct and modular C−H arylation of non‐substituted imidazo[2,1‐*b*]thiazole nucleus. With the support of methodological studies and DFT calculations, it was possible to disclose the different reactive positions, thus, to plan and develop two divergent transition metal‐promoted approaches for the selective arylation of the desired heterocycle.

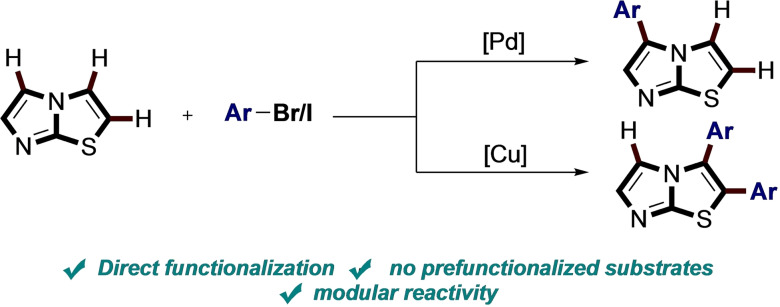

## Introduction

The heteroaromatic azole nuclei represent key structural features in a relevant number of compounds of significant synthetic interest. Their ubiquitous presence in biologically active substrates makes this class among the most explored chemical species in medicinal chemistry.^[1]^ In this context, imidazo [2,1‐b]thiazole (**1**) constitutes the core unit of a large cohort of biologically active species.^[2]^


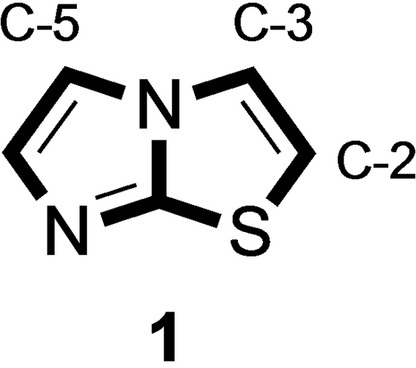


Since the late 60’s these compounds have been prepared, opportunely modified, and evaluated for potential biological activities.^[3]^ More recently, imidazo[2,1‐b] thiazole‐based drug candidates showed antiproliferative,^[4]^ anthelmintic^[5]^ (Levamisole and analogs),^[6]^ antifungal,^[7]^ antitubercular,^[8]^ antimicrobial,^[9]^ antioxidant activity^[10]^ as well as serotonin‐receptor ligands,^[11]^ also in combination with other bioactive compounds, upon appropriate structural modifications.[^4^, ^12^]

**Figure 1:**
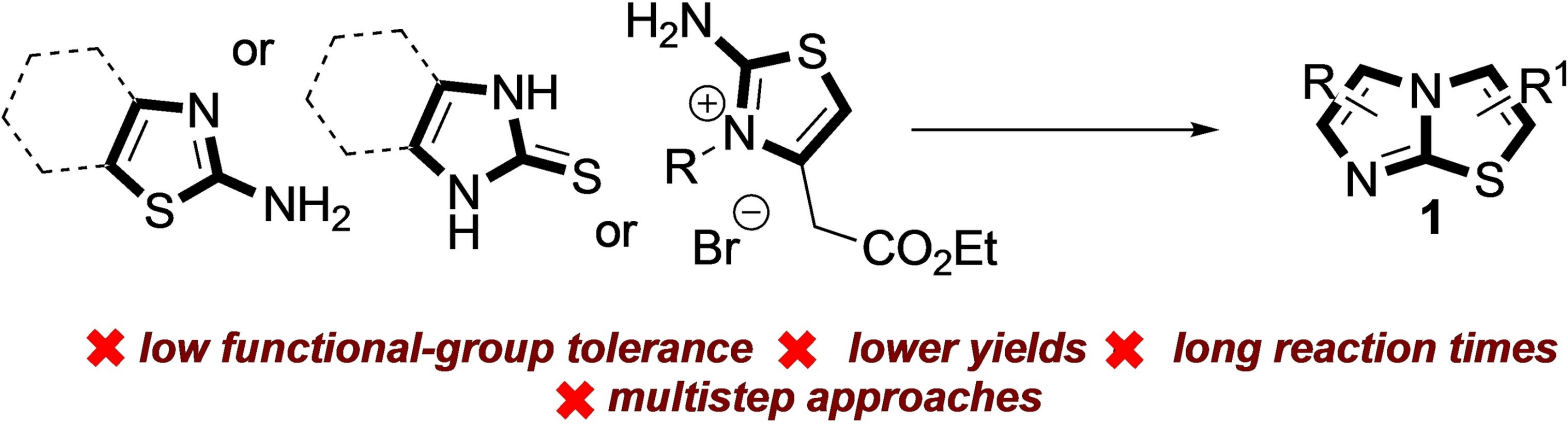
Stepwise approaches for the synthesis of functionalized imidazo[2,1‐b]thiazoles.

From a synthetic perspective, many different *de novo* approaches have been widely explored for the synthesis of imidazo[2,1‐b] thiazole derivatives, all based on the construction of its heteroaromatic bicyclic core (Figure 1).

However, these strategies often suffer from low functional‐group tolerance,[^3^, ^5^, ^13^] many synthetic steps[^4^, ^9^, ^14^] and, consequently, low overall yields.[^12^, ^15^]

**Figure 2:**
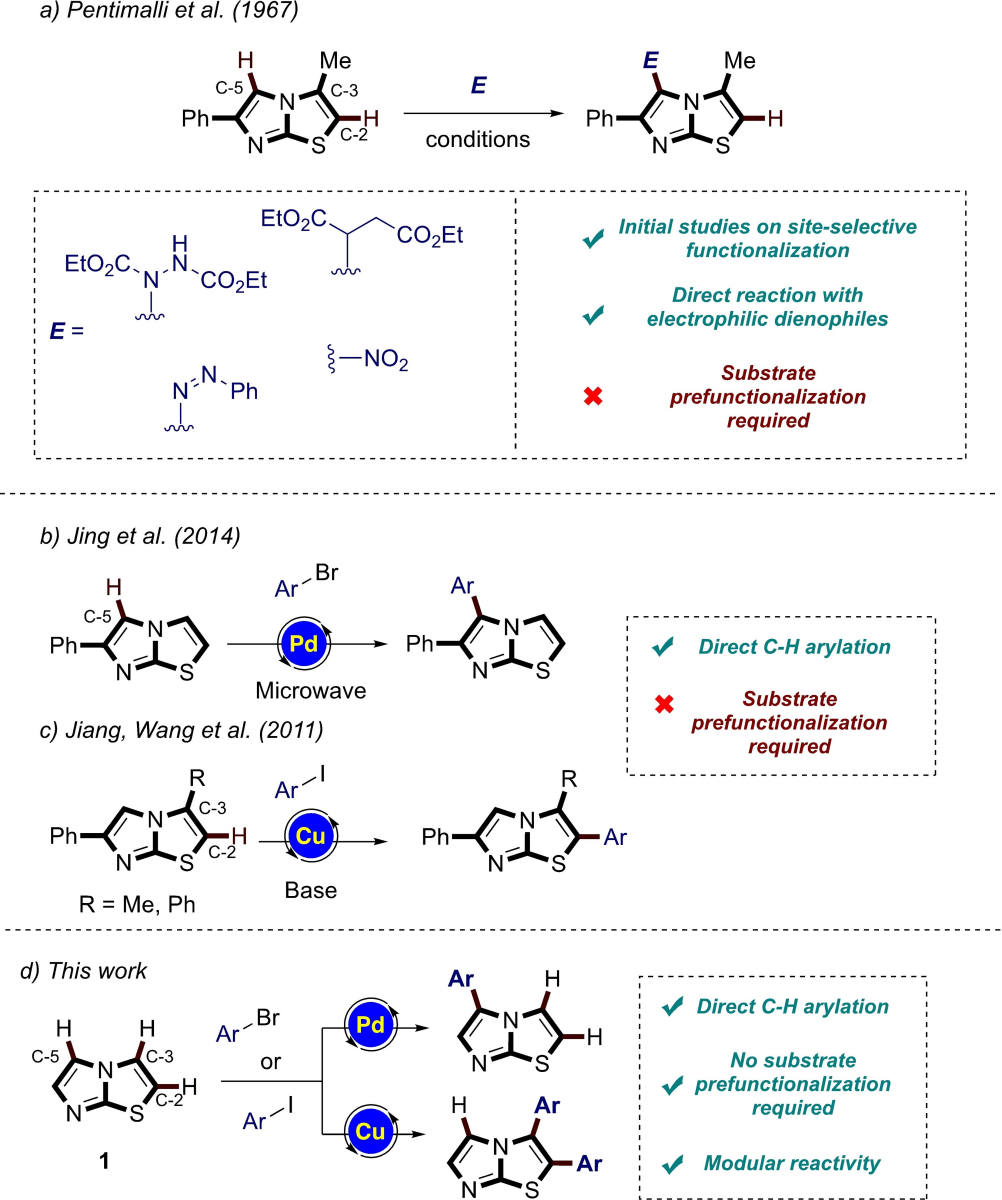
Previous work: a) Pentimalli's approach, b) and c) published direct C−H arylation procedures, d) this work.

Therefore, a direct and straightforward approach for the preparation of functionalized imidazo [2,1‐b] thiazoles is highly sought. Transition metal‐catalyzed C−H activation has nowadays consolidated its key role as an essential tool for synthetic organic chemists, thus allowing for direct functionalization of the desired scaffold, avoiding stepwise syntheses and the manipulation of air‐ sensitive reagents.^[16]^ In a series of studies devoted to the functionalization of the imidazo [2,1‐b] thiazole scaffold, in the late 60’s Pentimalli, Guerra and Taddei observed that position C‐5 reacts with electrophiles, while positions C‐2 and C‐3 remain substantially unaffected (Figure 2a).^[17]^

These pioneering works, albeit performed on 6‐aryl substituted imidazothiazoles, paved the way for the comprehension of the peculiar reactivity of the different positions. More recently, Jing and coworkers proposed a direct arylation of position C‐5 of 6‐aryl imidazo [2,1‐b] thiazoles through palladium catalysis under microwave irradiation (Figure 2b).^[18]^ On the other hand, Jiang, Wang and co‐workers reported on the copper‐mediated functionalization of position C‐2 of 3,6 disubstituted imidazo [2,1‐b] thiazoles (Figure 2c).^[19]^ These results suggest that the “imidazole” side of the bicyclic system has the most nucleophilic character, while the free C−H bond on the “thiazole” side (C‐2) is the most acidic one. It is in fact well known that the direct palladium‐catalyzed arylation of azoles with aromatic halides generally involves the most nucleophilic positions,[^17^, ^18^] while conducting the coupling in the presence of copper (I) salts results in a switch in selectivity, since the more acidic C−H bonds are generally affected.^[19]^

However, the results described so far (Figure 2a,b,c) are, to the best of our knowledge, uniquely based on the use as precursors of imidazothiazoles already substituted in one or more positions, therefore not allowing to evaluate the real efficiency of the reaction conditions both in terms of regioselectivity (being occupied one of the two positions on the “imidazole‐like” or “thiazole‐like” half), and in terms of mono‐*vs*. polyarylation. Moreover, as will be discussed later, the presence of aromatic substituents not only reduces the possibility of having mixtures of regioisomers or polysubstituted products, but also dramatically modifies the reactivity of the residual C−H bonds when compared with the behavior of the same bonds in unsubstituted **1**.

The peculiar structure of **1**, consisting essentially of a thiazole and an imidazole joined by the sharing of a nitrogen atom (nitrogen at position 1 in *N*‐substituted imidazoles, nitrogen at position 3 in thiazoles) and a carbon (at position 2 in both monocyclic azoles), combined with the absence of procedures for its direct arylation and the relevance of imidazothiazoles in medicinal chemistry, have tickled our scientific curiosity. Given our continued interest on the selective functionalization of azoles by activation reactions of aromatic C−H bonds,[^16^, ^20^] we could not fail to investigate the behavior of the four C−H bonds present in **1** in direct arylation reactions with aromatic halides, in the presence of transition metals. Our main objective was to identify conditions that could allow the selective functionalization of one of the two “halves” of **1**, the “imidazole” and “thiazole” sides, if both had maintained the typical reactivity of the two isolated azoles, as well as motivating what was experimentally found.

Herein, we are pleased to report the results of this investigation, which confirmed the hypothesis that in imidazothiazole **1** the two fused nuclei somehow retain the typical reactivity of *N*‐substituted imidazoles and thiazole.[^20^, ^21^] This allowed us to identify effective reaction conditions in promoting the selective arylation of one of the two halves simply by using a different transition metal as a coupling promoter (Figure 2d). Through DFT calculations we were also able to explain the experimental results, confirming the mechanistic reasons behind the different selectivity observed when the arylation was carried out in the presence of palladium or copper salts.

## Results and Discussion

### Pd‐Catalyzed C−H Arylation of Imidazo [2,1‐b] Thiazole (1)

We started our investigation on the viability of selective arylation of **1** by direct C−H activation by evaluating its behavior in the presence of a palladium pre‐catalyst. Considering the results reported by Jing and coworkers,^[18]^ who substantially applied the classical Miura's conditions for the selective C‐5 arylation of azoles^[22]^ to the selective C‐5 arylation of 6‐phenyl substituted imidazo [2,1‐*b*] thiazoles under microwave irrradiation,^[18]^ a first test was carried out by reacting **1** with 1‐bromo‐4‐nitrobenzene (**2** **a**) (1.5 equiv) as the coupling partner, in presence of palladium (II) acetate (Pd(OAc)_2_, 5 mol %) as the catalyst, triphenylphosphine (PPh_3_, 10 mol %) as the ligand, potassium carbonate (K_2_CO_3_, 2.0 equiv) as the base in dimethylformamide (DMF, 2.0 mL)) as the reaction solvent (entry 1, Table 1).

**Table 1:** General optimization conditions for C‐5 monoarylation.

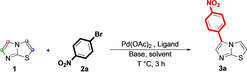					
Entry^[a]^	Ligand	Base	Solvent/T °C	Yield of **3 a** (Isolated)^[b]^	C5 Selectivity^[c]^
1^[d]^	PPh_3_	K_2_CO_3_	DMF/130	44	0.72
2	PPh_3_	K_2_CO_3_	DMF/140	54	0.85
3^[e]^	PPh_3_	K_2_CO_3_	DMF/140	41	0.85
4	PCy_3_	K_2_CO_3_	DMF/140	38	0.45
5	P(2‐furyl)_3_	K_2_CO_3_	DMF/140	61(56)	0.95
**6^[f]^**	**P(2**‐**furyl)_3_**	**K_2_CO_3_**	**DMF/140**	**47**	**0.94**
7	Dppb	K_2_CO_3_	DMF/140	39	0.71
8	Dppf	K_2_CO_3_	DMF/140	27	0.86
9	P(2‐furyl)_3_	K_2_CO_3_	DMF/120	66(61)	0.97
10	P(2‐furyl)_3_	KOAc	DMF/120	64	0.97
11	P(2‐furyl)_3_	K_3_PO_4_	DMF/120	54	0.94
12	P(2‐furyl)_3_	K_2_CO_3_	DMA/120	58(53)	0.95
13	P(2‐furyl)_3_	K_2_CO_3_	NMP/120	55(50)	0.95
14	P(2‐furyl)_3_	K_2_CO_3_	ACN/120	48	0.88
15	P(2‐furyl)_3_	K_2_CO_3_	*p*‐xylene/120	38	0.79
16	P(2‐furyl)_3_	K_2_CO_3_	GVL/120	40	0.90
17	P(2‐furyl)_3_	K_2_CO_3_	Me‐THF/120	29	0.83

[a] General conditions: **1** (0.5 mmol), *p*‐bromonitrobenzene (1.5 equiv), Pd(OAc)_2_ (5 mol %), ligand (10 mol %), base (2 equiv.), solvent (2 mL), at the reported temperature in a closed vessel under an argon atmosphere, unless otherwise reported. [b] Determined by UPLC‐MS‐DAD analysis, using 2‐phenylbenzoimidazole as internal standard; in parenthesis isolated yield of **3 a**. [c] C‐5 selectivity was expressed as the ratio between the area percent (AP %) of **3 a** in the UPLC chromatogram and the sum of the areas of all the three observed monoarylated imidazothiazoles. AP % values are uncorrected for the differences in UPLC response factors. [d] The arylation was carried out in a classical open vessel, under an argon atmosphere. [e] *p*‐Iodonitrobenzene (**4 a**) (1.5 equiv.) instead of **2 a** was used as the coupling partner. [f] The arylation was carried out under air.

After 24 hours at 130 °C the C‐5 monoarylated imidazothiazole **3** **a** was obtained in a 44 % UPLC yield (entry 1, Table 1). Two regioisomers of **3 a** (of the three possible) were identified in the crude reaction mixture by UPLC‐MS‐DAD analysis, resulting in a C‐5 selectivity of 0.72. This result contrasts with what was previously obtained when the same reaction was carried out using 6‐phenylimidazothiazole instead of **1**.^[18]^ In that case, in fact, the corresponding C‐5 arylated imidazothiazole was isolated in 72 % yield, and the presence of regioisomers was not described.^[18]^

**Figure 3:**
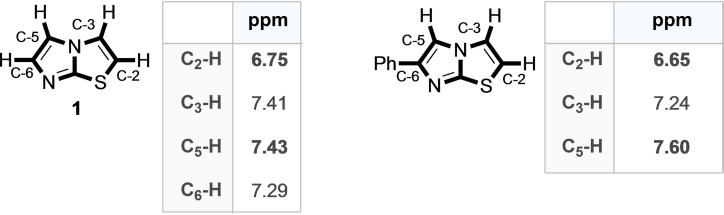
^1^H‐NMR chemical shifts of imidazothiazole **1** and its 6‐phenyl analogue. Values taken from Ref. [17c]

The presence of a benzene ring at position 6 on the imidazothiazole nucleus, therefore, has a significant effect on the reactivity of C−H bonds when compared with the reactivity of the unsubstituted nucleus of **1**. This is also well evidenced by simply examining the ^1^H‐NMR chemical shift values of the two nuclei (Figure 3).

In fact, the aromatic ring in position 6 has the consequence of better differentiating the three positions from an electronic point of view, shifting the signal relating to position 5 to lower fields, and that relating to positions 2 and 3 to higher fields (Figure 3). This, of course, makes it easier to identify conditions that lead to high regioselectivity, as well as better yield.

In our case, however, although the observed selectivity was not excellent, it confirmed that position C‐5 is the most reactive when a palladium‐based pre‐catalyst is used, validating the conclusions of Pentimalli and coworkers.^[17a]^ Noteworthy, the ”imidazole side” of **1** has an enhanced nucleophilic character compared to the “thiazole side”, while among position C‐5 and C‐6, it is the first to be the most reactive, if not the only one.^[18]^ Considering this first result, we therefore judged appropriate to continue the investigations by performing a more in‐depth screening, in order to improve yield and C‐5 selectivity of the direct arylation of **1**.

Interestingly, raising the reaction temperature up to 140 °C **3** **a** was obtained in a 54 % UPLC yield after 3 h, and a C‐5 selectivity of 0.85 was scored (entry 2, Table 1). Considering the enhanced reactivity of aryl iodides toward the arylation of electron‐rich azole nuclei, being more active in the oxidative addition step of the mechanistic pathway (see later),[^22^, ^23^] we hypothesized an increase of the reaction outcome by replacing the bromide derivative **2** **a** with 1‐iodo‐4‐nitrobenzene (**4** **a**). Unfortunately, a worse experimental result was obtained, and 5‐substituted imidazothiazole **3** **a** was obtained with a lower 41 % yield, albeit with a similar C‐5 selectivity (entry 3, Table 1), presumably due to partial catalyst poisoning.^[24]^ Having taken note of this result, we decided to verify the influence of the stereoelectronic nature of the palladium ligand on the outcome of the reaction.

Interestingly, while the replacement of PPh_3_ with the more basic and hindered tricyclohexylphosphine (PCy_3_) resulted in a drop in terms of monoarylation yield (38 %) and C‐5 selectivity (0.45) (entry 4, Table 1), the use of a metal ligand with diametrically opposed characteristics such as tri (2‐furyl) phosphine (P(2‐furyl)_3_) led to an UPLC yield of 61 % of **3** **a** (56 % isolated yield), and a satisfactory C‐5 selectivity of 0.95 (entry 5, Table 1). In contrast, when the coupling was carried out under aerobic conditions (entry 6, Table 1) **3** **a** was obtained in a lower 47 % yield, albeit with the same selectivity. Notably, the use of two typical bidentate phosphorous ligands such as 1,4‐bis (diphenylphosphino) butane (dppb) and ferrocenediyl‐bis (diphenylphosphine) (dppf) gave worse results in terms of **3** **a** yield and C‐5 selectivity (entries 7 and 8, Table 1).

Starting from the result described in entry 5, Table 1, further studies were then conducted on the temperature, types of base and solvents. Lowering the temperature to 120 °C resulted slightly beneficial: 61 % isolated yield and an almost complete C‐5 selectivity (0.97) (entry 9, table 1) was observed. The replacement of K_2_CO_3_ with other bases such as potassium acetate (KOAc) or potassium phosphate (K_3_PO_4_) did not lead to any significative improvement in yield and selectivity (entries 10 and 11, Table 1). To complete the methodological study, alternative solvents were tested instead of DMF. Interestingly, while DMA and NMP showed comparable outcomes (entries 12 and 13, Table 1), the use of acetonitrile (ACN),^[19]^ *p*‐xylene,^[18]^ or the biosolvents 2‐MeTHF and γ‐valerolactone (GVL)^[25]^ led to a dramatic drop in yield and selectivity (entries 14, 15, 16, and 17, Table 1).

**Scheme 1:**
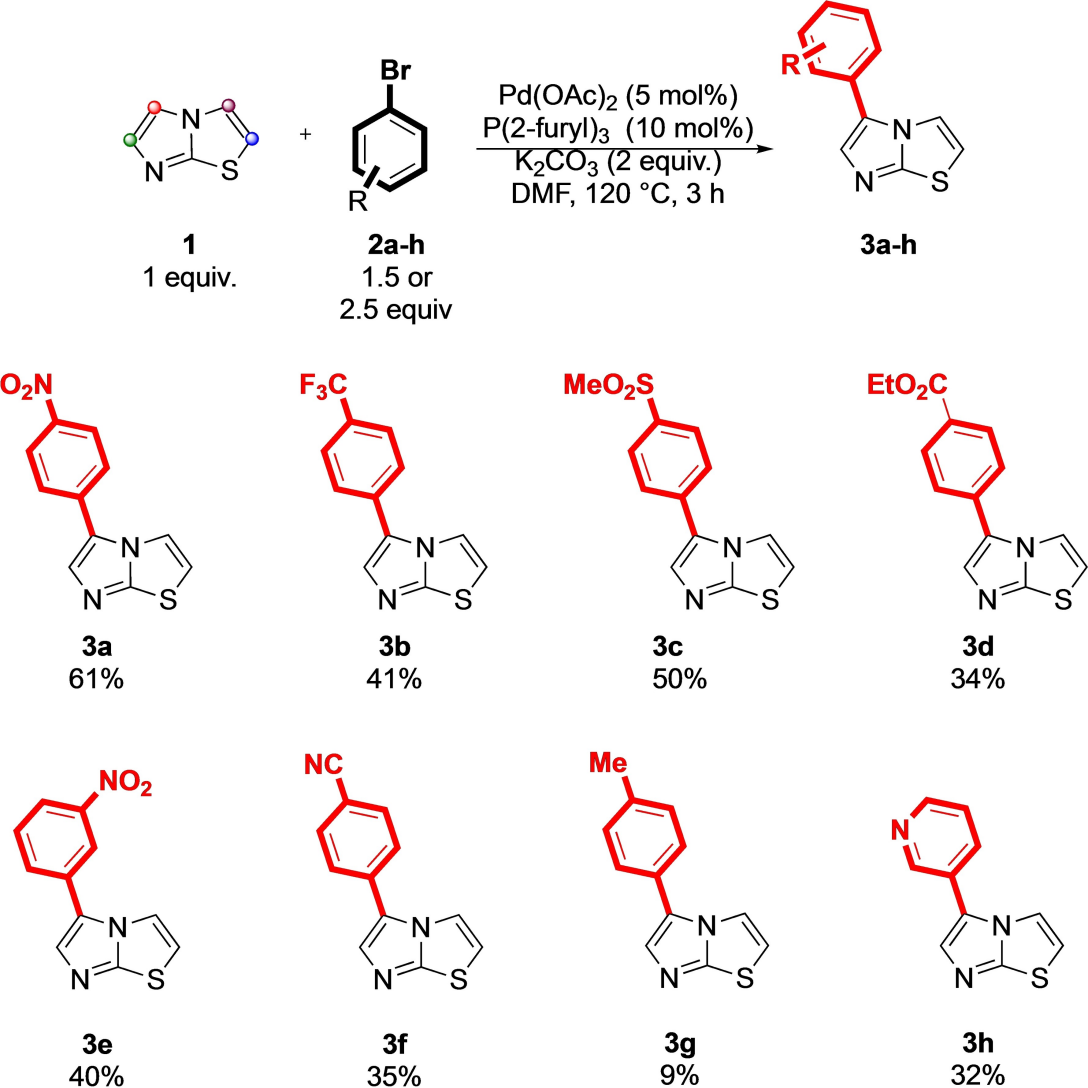
General representative scope for palladium‐catalyzed direct C‐arylation of imidazo[2,1‐b] thiazole (**1**) with (hetero) aryl bromides **2 a**–**h**.

With the optimized protocol in hand (entry 9, Table 1), we next investigated a representative viable scope for the palladium‐catalyzed transformation aiming to test, through the reaction between **1** and bromo (hetero) arenes if the established conditions would work efficiently (both in terms of yield and selectivity) for a selective C‐5 functionalization (Scheme 1).

In details, the coupling between **1** and 1‐bromo‐4‐(trifluoromethyl) benzene (**2** **b**) gave the monoarylated imidazothiazole **3** **b** in 41 % isolated yield. The reaction with other aromatic bromides bearing EWGs such as 1‐bromo‐4‐(methylsulfonyl) benzene (**2** **c**), ethyl 4‐bromobenzoate (**2** **d**), and 1‐bromo‐3‐nitrobenzene (**2** **e**) provided **3** **c**, **3** **d**, and **3** **e**, respectively, in 50 %, 34 %, and 40 % isolated yield, respectively. The reactivity of imidazo [2,1‐b] thiazole (**1**) was also tested with 4‐bromobenzonitrile (**2** **f**) and 1‐bromo‐4‐methylbenzene (**2** **g**). In the first case, the required 5‐arylimidazothiazole **3** **f** was obtained in 35 % isolated yield, while the electron‐rich bromide **2** **g** provided the expected **3** **g** in a a poor a 9 % yield. This last negative result is, unfortunately, in accordance with the results reported for 6‐phenylimidazothiazole. Also in that case, in fact, the introduction of EDGs resulted in a dramatic loss of the coupling efficiency.^[18]^ Derivative **3** **h** was finally obtained in 32 % yield by reacting **1** with 3‐bromopyridine (**2** **h**). As general consideration, we were pleased to confirm the adaptability of the protocol toward differently substituted aryl halides, observing experimentally the superior nucleophilic character of the C‐5 over the other positions through palladium catalysis conditions. This evidence emerges even without the presence of substituents in position C‐6 and C‐3 of the imidazo [2,1‐b]thiazole core.[^17^, ^18^]

### Cu‐Mediated C−H Arylation of Imidazo[2,1‐b]Thiazole (1)

Given the predictable difference in reactivity between the C−H bonds of 1 based on the NMR data (Figure 3) and proved that the C5‐H bond of **1** is the most reactive in palladium‐catalyzed direct arylation reactions, the next step was to identify suitable conditions for the selective arylation of the C‐2/C‐3 positions.

Experimental evidence of the difference in reactivity of positions C‐2 and C‐3 of imidazothiazoles when compared with C‐5 was reported as early as 1967. In fact, Pentimalli and co‐workers found that while the reaction of 6‐phenylmidazothiazole and 3‐methyl‐6‐phenylimidazothiazole with maleic anhydride led to the isolation of the substitution product at position C‐5, when the same reaction was carried out on 5‐methyl‐6‐phenylmidazothiazole, no formation of any product is observed, even if positions 2 and 3 are free.^[17b]^ In contrast, in a series of papers devoted to Mannich‐type reactions involving 6‐aryl‐3‐methylimidazothiazole derivatives, Han and co‐workers observed a selective C2‐H deprotonation by treating 1 with LDA at low temperature, thus experimentally highlighting the higher kinetic acidity of the C2‐H bond compared to the C5‐H bond.^[26]^

As regards direct C−H arylation, in 2011 Wang and coworkers described a protocol for the direct C‐2 arylation of 3,6‐disubstited imidazothiazoles using a catalytic amount of copper (I) chloride (CuCl), in the presence of lithium *tert*‐butoxide (*t*‐BuOLi) (Figure 3c).^[19]^ Four years later, the same research group proved that the coupling could be mediated also by copper (II) salts, such as CuCl_2_, without changing the C2 selectivity.^[27]^

Considering these results, we decided to evaluate whether the use of copper salts could effectively allow a switch in selectivity, thus leading to a selective arylation of the thiazole half of **1**. As a first test, we decided to verify whether reaction conditions very similar to those developed by Wang and coworkers in 2011 for the arylation of 3‐methyl‐5‐phenylmidazothiazole^[19]^ could also be successfully applied to selective arylation of **1**. Thus, imidazothiazole **1** (0.5 mmol) was reacted with 4‐bromotoluene (1.5 equiv) (**2** **f**), chosen as a typical aryl bromide, in the presence 20 mol % of copper (I) iodide (CuI) and 2.0 equiv of lithium tert‐butoxide (*t*‐BuOLi) in DMA. Unfortunately, after 24 h at 140 °C a UPLC‐MS‐DAD analysis of the resulting reaction mixture revealed the presence of mono‐ and diary‐based products only in trace amounts, along with with the disappearance of precursor **1** (entry 1, Table S1).

Assuming that this negative result could be attributed to the use of a strong base such as *t*‐BuOLi, we decided to continue the investigation by evaluating the use of weaker bases. Considering the results reported by Miura and collaborators on the use of carbonates in copper (I)‐promoted direct arylations of *N*‐methylimidazole and thiazole,^[22]^ we then carried out a second test using 2.0 equiv of potassium carbonate (K_2_CO_3_) and 2.0 equiv of CuI in DMA at 140 °C for 48 h. As summarized in entry 1, Table 2, the use of carbonate as the base led to a mixture of four products: the monoarylated 2‐(*p*‐tolyl) imidazo[2,1‐*b*]thiazole (**5 a**) and 3‐(*p*‐tolyl) imidazo [2,1‐*b*] thiazole (**6 a**), in a 59/41 UPLC ratio, along with the diarylated 2,3‐di‐*p*‐tolylimidazo [2,1‐*b*] thiazole (**7 a**) and 2,5‐di‐*p*‐tolylimidazo [2,1‐*b*]thiazole (**8 a**), in a 85/14 UPLC ratio. This result, although confirming the higher kinetic acidity of the C2‐H and C3‐H bonds compared to the C5‐H bond of **1**, shows that the difference in reactivity of the two positions on the thiazole portion is not sufficient to allow a selective monoarylation at the C2 position. It is worth mentioning that the introduction of the first aryl group certainly influences the reactivity of the adjacent position. It must be considered also that in direct arylation reactions of 1,3‐azoles the use of copper salts, in the presence or absence of a palladium co‐catalysis, usually lead to a switch in the selectivity when only a palladium catalyst is used, allowing a selective functionalization of the position that has the most acidic C−H bond. The numerous studies following the results published in the seminal paper by Miura and coworkers in 1998.^[22]^ have made it possible to highlight that one of the roles of copper is the coordination with azoles to form π‐complexes. This aspect has as a consequence the lowering of the pKa of their acidic C−H bonds, thus increasing their innate reactivity.[^20^, ^28^]

**Table 2:** General optimization conditions for C‐2/C‐3 diarylation.

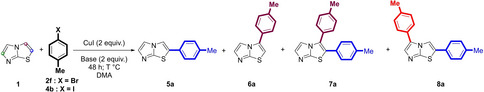								
Entry^[a]^	X (equiv)	Base	T (°C)	Conversion of **1** ^[b]^	**5 a**/**6 a** AP % ratio^[c]^	**7 a**/**8 a** AP % ratio^[c]^	Mono/diarylated product ratio (AP %)	Product(s) isolated yield (%)
1	Br (1.5)	K_2_CO_3_	140	67	59/41	93/7	85/14	–
2	Br (4.0)	K_2_CO_3_	140	95	55/45	82/18	38/62	13(**5** **a**), 11(**6** **a**), 49(**7** **a**), 10(**8** **a**)
3	Br (4.0)	K_2_CO_3_	160	>99	54/46	89/11	16/84	68(**7** **a**)
4^[d]^	Br (4.0)	K_2_CO_3_	160	93	57/43	90/10	22/78	51(**7** **a**)
5	I (4.0)	Cs_2_CO_3_	140	98	58/42	91/9	9/91	55(**7** **a**)
**6**	**I** (**4.0**)	**Cs_2_CO_3_**	**160**	**>99**	**55/45**	**95/5**	**4/96**	85(**7** **a**)
7^[e]^	I (4.0)	Cs_2_CO_3_	160	80	51/49	94/6	10/90	62(**7** **a**)

[a] General conditions: **1** (0.5 mmol), **2** **f** or **4** **b** (1.5 or 4.0 equiv, see Table), base (2.0 equiv), CuI (2.0 equiv), DMA (2 mL), 140 or 160 °C, 48 hours, unless otherwise reported. [b] Determined by GLC‐FID analysis, using biphenyl as internal standard. [c] The **5** **a**/**6** **a** ratio (i. e. C2/C3) and the **7** **a**/**8** **a** (i. e. C2,3/C2,5) ratio were expressed as the ratio between the area percent (AP %) of the compounds in the UPLC‐MS‐DAD chromatogram. AP % values are uncorrected for the differences in response factors. [d] The reaction was carried out for 72 hours. [e] The coupling was carried out using 0.2 equiv of CuI for 72 h.

The formation of the diaryl‐substituted imidazothiazoles **7** **a** and **8** **a** derivatives was not, however, entirely unexpected, since previously published results on copper‐catalyzed arylations of imidazothiazoles had been carried out only on derivatives having the C3 position occupied,[^19^, ^27^] and that the main products of the copper‐promoted reactions involving imidazole and thiazole were those of diarylation.^[22]^

Given the result summarized in entry 1 of Table 2, that suggested the possibility of selectively obtaining none of the monoarylation products **5** **a** or **6** **a**, but that of diarylation **7** **a**, we decided to evaluate at first the influence of weak bases other than carbonate on the outcome of the reaction.

Disappointingly, the use of potassium phosphate (K_3_PO_4_) or potassium acetate (KOAc) resulted in a drop both of conversion and selectivity, also confirming the very similar reactivity of positions C2 and C3 (entries 4 and 5, Table S1).

Acknowledging the impossibility of obtaining a selective monoarylation but considering instead that it might be possible to develop a selective and efficient copper‐mediated C‐2/C‐3 diarylation leading to imidazothiazoles of general formula **7**, a test using 4 equiv of **2** **f** was then carried out.

As we hoped for, we were able to isolate **7** **a** in a 49 %, yield (entry 2, Table 2). By means of a thorough purification of the crude reaction mixture by flash chromatography we isolated, for the sole purpose of their structural confirmation, the monoarylated derivatives **5** **a** and **6** **a**, and 2,5‐diarylimidazothiazole **8** **a** (entry 2, Table 2). An increase in reaction temperature up to 160 °C further increased the yield of **7** **a** to 68 % with a clean, but not yet satisfactory, enhancement of the monoarylation/diarylation ratio up to 16/84, (entry 3, Table 2). On the other hand, maintaining the reaction for a longer time did not improve the outcome of the reaction (entry 4, Table 2).

A gain in terms of selectivity was instead observed when bromo‐4‐methylbenzene (**2** **f**) was replaced with the analogous iodo‐4‐methylbenzene (**4** **b**). Inspired by the previous reports on the use of cesium‐based bases when (hetero) aryl iodides are the partners in direct arylations of azoles,[^22^, ^29^] **1** and **4** **b** were reacted in the presence of cesium carbonate (Cs_2_CO_3_) instead of K_2_CO_3_. As shown by the results summarized in entries 5, 6, and 7 of Table 2, the use of an aromatic iodide combined with a cesium salt led to excellent results. In fact, if conducting the reaction at 140 °C gave **7** **a** in a 55 % isolated yield (entry 5, Table 2), simply raising the reaction temperature to 160 °C allowed us to isolate **7** **a** in a satisfactory 85 % isolated yield (entry 6, Table 2). Finally, it must be noted that the double direct arylation of **1** may be promoted also by a catalytic amount of CuI (entry 7, Table 2), but 72 h instead of 48 h were required to obtain a reasonable conversion, and a lower yield of **7** **a** was indeed obtained (compare entries 6 and 7, Table 2).

The good result obtained in the preparation of **7** **a** from **1** and **4** **b** under the experimental conditions reported in entry 6, Table 2 prompted us to extend this new procedure to the selective preparation of 2,3 diaryl‐substituted imidazothiazoles **7** **b**–**g**.

**Scheme 2:**
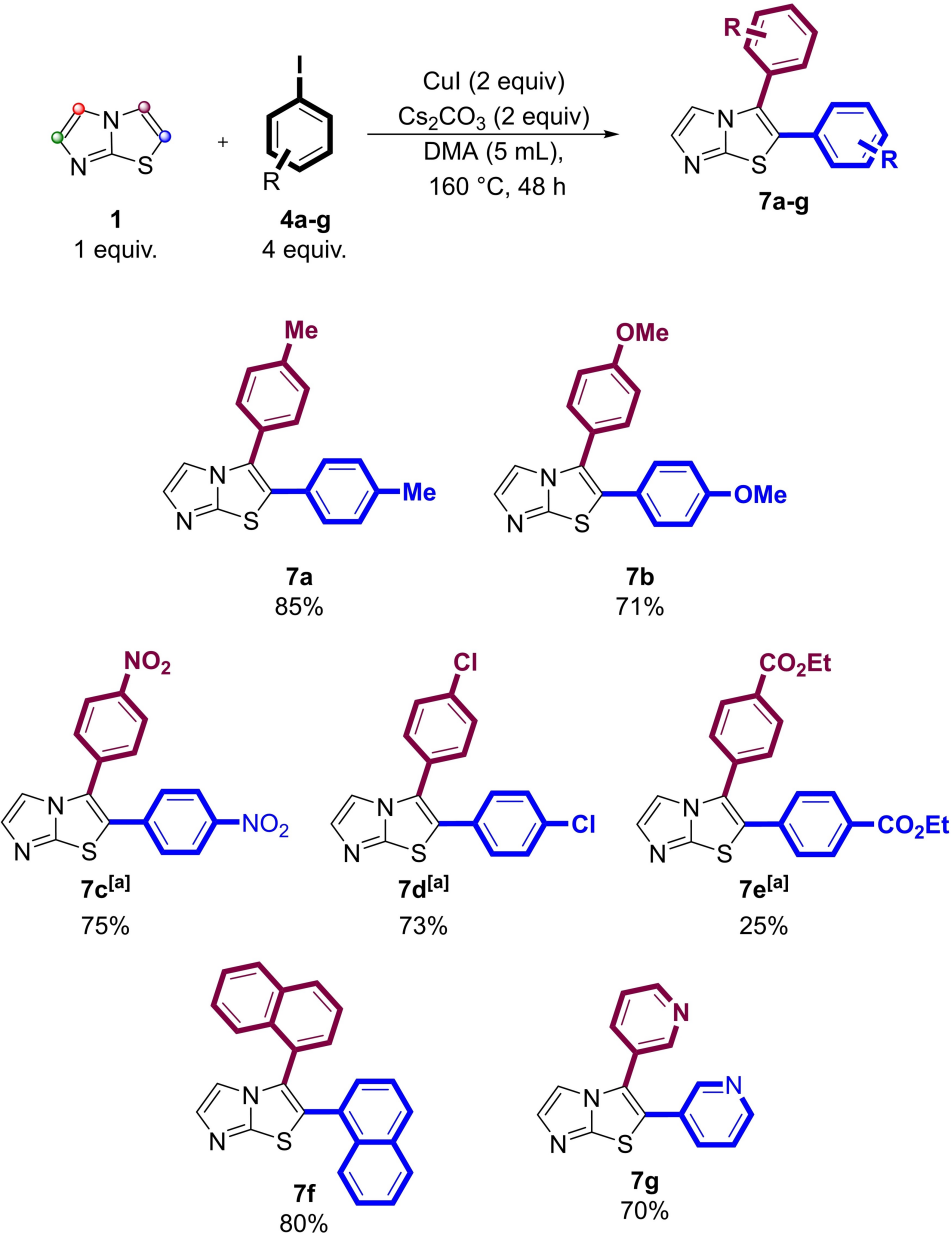
General scope for the selective C‐2/C‐3 diarylation of imidazo[2,1‐b] thiazole (**1**); [a] 48 hours reaction.

Scheme 2 summarizes the results obtained in these highly selective reactions, which generally gave the required 2,3‐diarylheteroarenes in high yields. Notably, this copper (I)‐promoted protocol for direct di‐arylation of **1** gave in general high yields regardless of the electronic nature of the aromatic iodide or its steric hindrance.

In fact, compounds **7** **c** and **7** **d**, bearing a typical EWG groups, were obtained in 75 % and 73 % isolated yield, respectively, while imidazothiazoles **7** **a** and **7** **b** bearing EDG groups were obtained in 85 % and 71 %, isolated yield, respectively. Unfortunately, the reaction between **1** and ethyl 4‐iodobenzoate (**4** **e**) resulted in a significant degradation of the coupling partner and, therefore, to the isolation of the desired product **7** **e** in 25 % yield. The coupling of 1‐iodonaphthalene (**4** **f**), an example of sterically bulky aryl moiety, with **1** led to the expected double arylation product **7** **f** in 80 % isolated yield. Finally, the use of 3‐iodopyridine (**4** **g**) as an example of heteroaromatic iodide led to the isolation of the desired imidazothiazole **7** **g** in a remarkable 80 % yield.

### DFT Studies and Mechanistic Proposal

**Figure 4:**
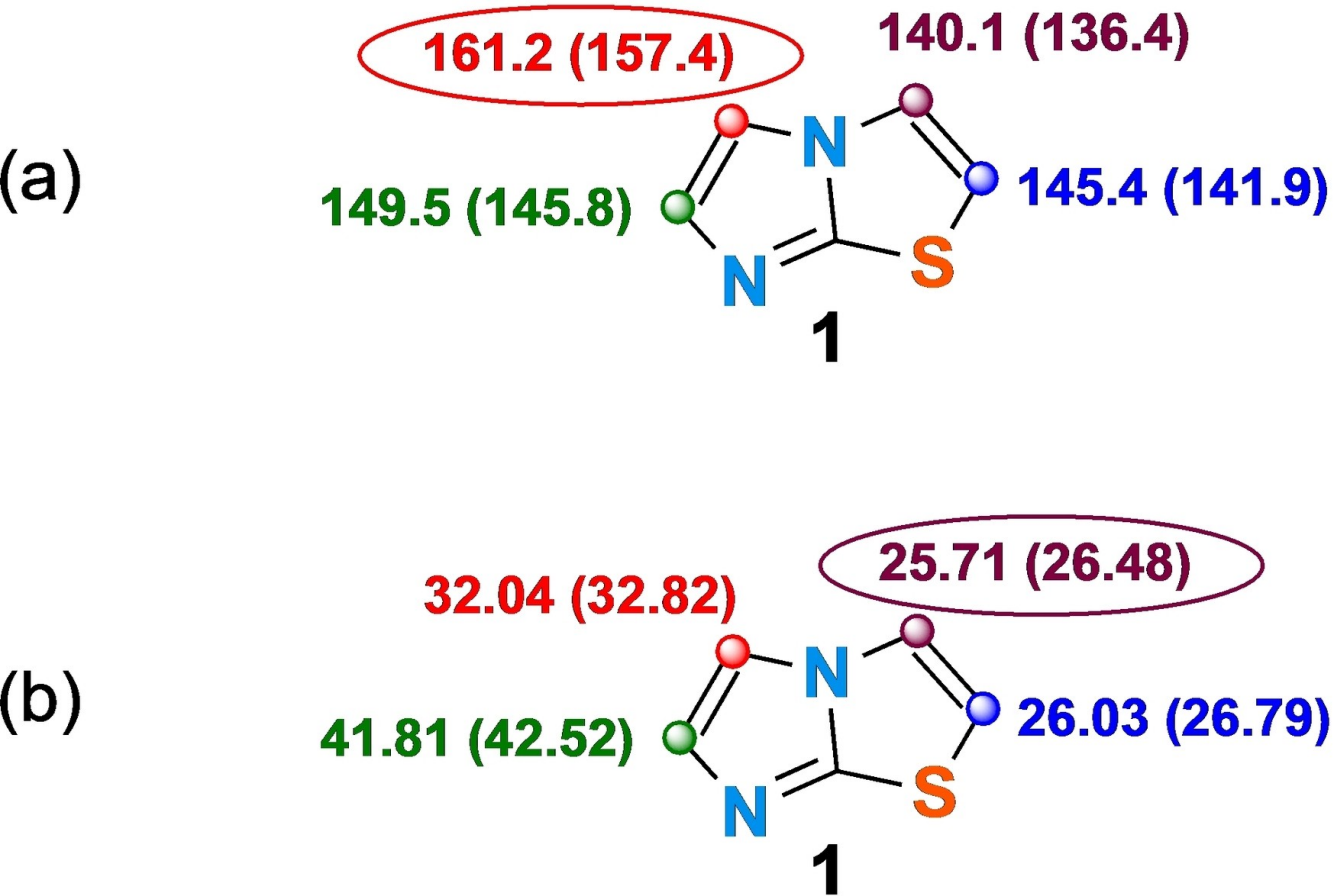
Summary of DFT computational results. a) Calculated (B3LYP/6‐311++G(2df,2pd)+SMD) free energies (kcal/mol) of the electrophile affinity (Eα) of imidazothiazole **1** by Br^+^ cation in DMA at 25 °C and, in parenthesis, at 160 °C b) Calculated (B3LYP/6‐311++G(2df,2pd)+SMD) free energies (kcal/mol) for the deprotonation of each of each of the CH groups of imidazothiazole **1** by acetate in DMA at 25 °C and, in parenthesis, at 160 °C.

It is clear from previous reports that the natural site of electrophilic substitution of imidazothiazoles is their C5 position,[^17^, ^18^] while C2‐H is the most acidic site, at least in 3,6‐disubstituted imidazothiazoles.^[26]^ Our experimental results, which confirmed what has already been reported regarding the reactivity of position C‐5 but evidenced a comparable reactivity of positions C‐2 and C‐3, have been fully supported by density functional theory (DFT) calculations, performed using DMA as the solvent at 25 °C and 160 °C.^[30]^ These studies highlighted, in particular, the clear difference between the C‐5 position, that showed the highest electrophilic affinity (Eα) value (Figure 4a),^[31]^ and positions C‐2 and C‐3, that are the most acidic as can be seen from the comparison of the relative deprotonation energies (differing from each other slightly) (Figure 4b).^[32]^

As far as the reaction mechanism is concerned, experimental data and DFT calculations show an almost complete dependence of selectivity on the nature of the transition metal used.

**Figure 5:**
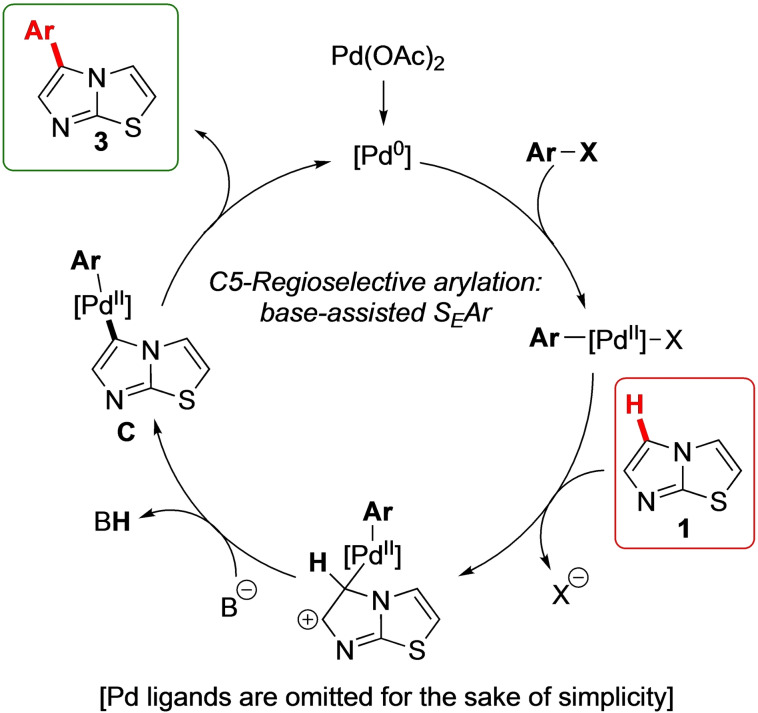
Proposed mechanism for the regioselective Pd‐catalyzed C5 direct arylation of imidazothiazole **1**.

Specifically, when the direct arylation of **1** is conducted under palladium catalysis, the most reactive site is the one with the highest electron affinity Eα. And, therefore, it is plausible to admit that in this case the C−H activation occurs by a base‐promoted electrophilic palladation (S_E_Ar) (Figure 5),[^22^, ^29^, ^33^] also considering that the most effective ligand resulted the poor σ‐donor P(2‐furyl)_3_,^[34]^ and the low yield observed when an electron‐rich aromatic bromide such as 4‐bromotolueme (**2** **a**) was employed.

**Figure 6:**
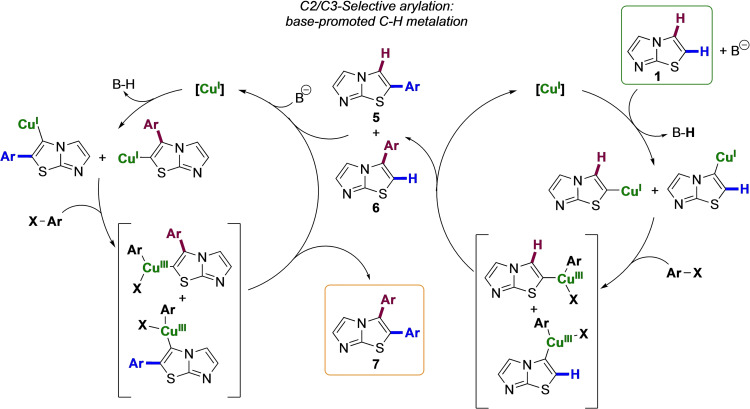
Proposed mechanism for the regioselective Cu‐promoted C2/C3 direct diarylation of imidazothiazole **1**.

On the other hand, the selective double arylation involving C2‐H and C3‐H might be rationalized supposing the mechanistic ptahway summarized in Figure 6. in accordance with previous reports,[^19^, ^35^] the double arylation pathway could be initiated by base‐promoted C−H metalation involving acidic C2‐H or C3‐H bonds. At higher temperatures, as also highlighted by DFT calculations, the acidity of these two positions does not differ enough to discriminate in which position the metalation comes first.^[36]^ It is also worth mentioning that the high C‐2/C‐3 selectivity observed in the presence of CuI can be rationalized taking into account the ability of the Cu(I) salts to metalate the most acidic C−H bonds of azoles.[^22^, ^37^]

The metalation step is than followed by a Cu(I)−Cu(III) oxidative addition‐reductive elimination sequence, giving rise to the monoarylated imidazothiazoles **5** and **6** that, still possessing sufficiently acidic C−H bonds, may be further involved in a second catalytic cycle to give the desired 2,3‐diarylated imidazothiazoles **7** (Figure 6).

As shown in Figure 6, the C‐2/C‐3 double copper‐mediated arylation reaction of imidazothiazole **1** should involve the use of a catalytic amount of CuI. However, to obtain reasonable reaction rates and satisfactory yields the reactions were carried out using a molar excess of this salt (compare entries 6 and 7 of Table 2). It is likely that this excess is necessary to facilitate the formation of significant amount of the imidazothiazole‐copper (I) compounds from neutral imidazothiazoles **1**, **4** and **5** (Figure 5).

## Conclusions

In conclusion, the present work aims to outline the feasibility of tuning the reactivity of unsubstituted imidazo [2,1‐b] thiazole **1** for a direct access to Csp^2^−H arylation products. Experimental and computational DFT studies revealed how the different positions behave toward divergent reaction conditions, thus providing the desired product of arylation in a selective manner, without recurring to tedious and low‐yielding stepwise approaches with the assistance of substrate pre‐functionalization. In the specific, C‐5 position shows enhanced reactivity toward electrophilic species, being capable of reacting, under palladium catalysis, providing a single product of monoarylation with satisfactory selectivity. Positions C‐2 and C‐3, less differentiable between each other, display a more acidic character and react under copper‐mediated conditions to provide C‐2/C‐3 diarylation products exclusively on the “thiazole‐side”. Further investigation into their potential applications in pharmaceuticals and as new organic materials are underway.

## Supporting Information

The authors have cited additional references within the Supporting Information.[^38^, ^39^]

## Conflict of Interests

The authors declare no conflict of interest.

1

## Supporting information

As a service to our authors and readers, this journal provides supporting information supplied by the authors. Such materials are peer reviewed and may be re‐organized for online delivery, but are not copy‐edited or typeset. Technical support issues arising from supporting information (other than missing files) should be addressed to the authors.

Supporting Information

## Data Availability

The data that support the findings of this study are available in the supplementary material of this article.
