# Supplementary material for: Transition Metal‐Driven Selectivity in Direct C−H Arylation of Imidazo[2,1‐b]Thiazole
Source: ChemistryOpen. 2024 Jul 25;13(11):e202400180. doi: 10.1002/open.202400180 (PMC12056928; doi:10.1002/open.202400180)
Supplement: Supplementary file 1 — Supporting Information [file OPEN-13-e202400180-s001.pdf]

ChemistryOpen

Supporting Information

Transition Metal-Driven Selectivity in Direct C—H Arylation of Imidazo[2,1-*b*]Thiazole

Antonio Del Vecchio, Elisabetta Rosadoni, Lorenzo Ballerini, Angela Cuzzola, Filippo Lipparini, Paolo Ronchi, Sara Guariento, Matteo Biagetti, Marco Lessi, and Fabio Bellina*

Transition Metal-driven Selectivity in Direct C–H Arylation of Imidazo[2,1-*b*]thiazole

A. Del Vecchio, E. Rosadoni, L. Ballerini, A. Cuzzola, L. Lipparini, M. Lessi, F. Bellina*

Department of Chemistry and Industrial Chemistry, Università di Pisa, Via Giuseppe Moruzzi, 13, 56124, Pisa, Italy. E-mail: fabio.bellina@unipi.it

P. Ronchi, S. Guariento, M. Biagetti, Chemistry Research and Drug Design, Chiesi Farmaceutici S.p.A, 43122, Parma Italy

Table of contents

General information ... *3*

General procedure and substrate scope for C-5 arylation of imidazo[2,1-b]thiazoles (3a-h) ... *4*

Screening of the reaction conditions for the direct C2/C3 arylation of 1 with 4-bromotoluene (2f) or 4-iodotoluene (4b) ... *7*

2-(p-tolyl)imidazo[2,1-*b*]thiazole (5a), 3-(p-tolyl)imidazo[2,1-*b*]thiazole (6a), 2,5-di-p-tolylimidazo[2,1-*b*]thiazole (8a) (entry 2, Table 2) ... 7

General scope for C-2 and C-3 diarylation of imidazo[2,1-b]thiazoles (7a-g) ... *12*

DFT Calculations ... *15*

Affinity towards electrophiles ... 15

Affinity towards electrophiles: imidazo[2,1-*b*]thiazole – Cu complex ... 16

Deprotonation ... 17

HOMO vs Potential ... 18

NMR spectra ... *19*

References ... *35*

General information

Melting points were recorded on a hot-stage microscope (Reichert Thermovar). Precoated silica gel PET foils (Sigma-Aldrich) were used for TLC analyses. Direct arylation reactions in closed vessels were carried out using Microwave 50 ® apparatus by Anton Parr. GLC-FID analyses were performed on a Dani GC 1000 chromatograph equipped with a PTV injector, using an Agilent J&W DB-1 column (15m x 0.25mm x 0.25μm) and recorded with a Dani DDS 1000 data station and a Shimadzu Nexis GC-2030 using an Agilent J&W DB-5 column (30 m x 0.25 mm i.d. x 0.25 μm). GLC-MS analyses were recorded with an Agilent 6890N gas chromatograph interfaced with an Agilent MS5973 mass detector, using an Agilent J&W DB-5ms (30m x 0.25mm x 0.25μm) column. LC-MS-DAD analyses were performed on an Acquity UPLC Water instrument (Phase A 95/5 H2O / ACN + 0.1% Formic Acid, Phase B 5/95 H2O / ACN + 0.1% Formic Acid; Acquity UPLC 2.1x100 mm column, BEH C18, 1.7 μm; Flow 0.5mL/min) coupled with an Acquity QDa Water mass spectrometer (Probe temperature: 600 °C; ESI capillary voltage 1.5kV; Cone voltage 15V; Mass range 60-1000 Da) and a PDA eλ Detector (wavelength range 200-800 nm). C-5 arylation methodology and substrates **3a,h** were prepared using a Monowave 50® reactor purchased by Anton Parr. Purifications by flash chromatography were performed using Merck 60 silica gel. Elemental analyses were acquired with an Elementar Vario Micro Cube in CHNS mode. ^{1}H-NMR, ^{13}C-NMR and ^{19}F-NMR spectra were recorded at 400, 100 and 376 MHz, respectively with a Jeol 400 spectrometer and at 500 and 125 MHz, respectively with a Jeol 500 spectrometer, referring chemical shifts to TMS or residual solvent signal. The following notation was used to report NMR spectra: s = singlet, bs = broad singlet, d = doublet, dd = double doublet, ddd = double, double doublet, t = triplet, dt = double triplet, q = quadruplet. All the commercially available reagents and solvents were used as received.

Screening of the reaction conditions for the direct C5 arylation of 1 with 1-bromo-4-nitrobenzene (2a)

A mixture of **1** (63 mg, 0.5 mmol), p-bromonitrobenzene (**2a**) (152 mg, 0.75 mmol, 1.5 equiv.), Pd(OAc)2 (5.6 mg, 0.025 mmol, 5 mol%), ligand (10 mol% if monodentate, 5 mol% if bidentate), base (1 mmol, 2 equiv.) in the selected solvent (2 mL), was stirred in a closed vial for Monowave 50®, maintained under argon for 3h at the selected temperature. After cooling to room temperature, the crude was diluted in DCM (5 mL) and stirred for 10 minutes; then, it was filtered over celite, and the filter was washed with acetone (40 mL) and DCM (40 mL). 2-Phenylbenzimidazole was added as

internal standard, and the resulting solution was analysed by UPLC-MS-DAD. Table 1 summarizes the results of this screening.

5(4-nitrophenyl)imidazo[2,1-*b*]thiazole (3a)

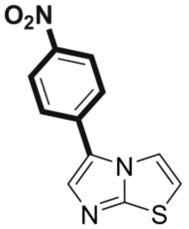

The crude obtained by the reaction of **1** with p-bromonitrobenzene (**2a**), (Table 1, entry 5) was purified by flash chromatography on silica gel using a mixture of toluene/iPrOH (90:10) as eluent to give **3a** (68 mg, 56% yield) as a bright yellow solid. M.p.=248-250 °C (dec). ^{1}H NMR (400 MHz, DMSO-d_6) δ: 8.38 (d, *J* = 4.48 Hz, 1H), 8.28 – 7.97 (m, 4H), 7.92 (d, *J* = 1.2 Hz, 1H) 7.53 (dd, *J* = 4.5 Hz, *J* = 1.2 Hz, 1H). ^{13}C NMR (100 MHz, DMSO-d_6) δ: 152.3, 145.4, 135.6, 135.5, 125.4, 125.0, 125.4, 119.7, 115.1. ESI-MS (+), m/z: 246 $[M+H]^+$. EI-MS, m/z (%): 245 (100), 215 (25), 199 (27), 198 (31), 187 (6), 172 (7), 141 (7). Elemental analysis: $C_{11}H_7N_3O_2S$ (245.03): calcd. C, 53.87; H, 2.88; N, 17.13; S, 13.07 found C, 54.05; H, 2.89; N, 17.76; S, 13.12.

This compound was also obtained in isolated yields of 61%, 53% and 50% as reported in Table 1, entries 8, 11 and 12.

General procedure and substrate scope for C-5 arylation of imidazo[2,1-*b*]thiazoles (3a-h)

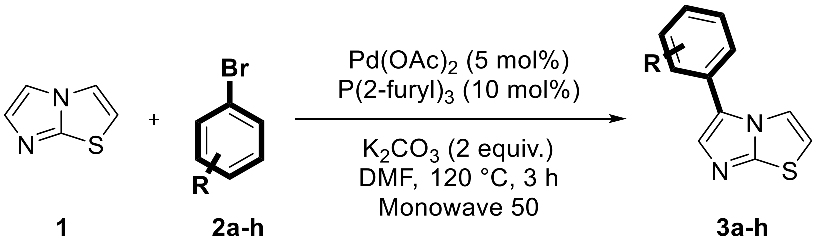

1 1 equiv. **2a-h** 1.5 equiv. **3a-h**

In a closed vial for Monowave 50®, solid reagents were sequentially added: imidazothiazole **1** (63 mg, 0.5 mmol, 1 equiv.), aryl-bromide (0.75 mmol, 1.5 equiv.), $Pd(OAc)_2$ (5.6 mg, 0.025 mmol, 5 mol%), $P(2\text{-furyl})_3$ (11.6 mg, 0.05 mmol, 10 mol%), K_2CO_3 (138.2 mg, 1 mmol, 2 equiv). Three *vacuum*-argon cycles were made and previously degassed DMF (2 mL) was added *via* syringe. The vial was sealed and loaded into the Monowave 50®, setting the reaction temperature at 120 °C and time at 3 hours. After cooling to room temperature, the crude was diluted with DCM (5 mL) and stirred for 10 minutes, filtered over Celite® and washed with acetone (30 mL) and DCM (30 mL). The solution was concentrated under reduced pressure and the residue was purified by flash chromatography on silica gel.

5-(4-(trifluoromethyl)phenyl)imidazo[2,1-*b*]thiazole (3b)

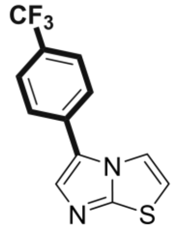

The crude was purified by flash chromatography on silica gel using a mixture of petroleum ether/EtOAc (45:55) as eluent to give **3b** (55 mg, 41% yield) as a beige solid. M.p.=116-118 °C. ^{1}H NMR (400 MHz, $CDCl_3$) δ: 7.72 – 7.71 (m, 2H), 7.70 (d, *J* = 4.5 Hz, 1H), 7.64 – 7.62 (m, 2H), 7.54 (d, *J* = 1.1 Hz, 1H), 6.97 (dd, *J* = 4.5 Hz, *J* = 1.1 Hz, 1H). ^{13}C NMR (100 MHz, $CDCl_3$) δ: 151.7, 133.8, 133.5, 129.4 (q, *J* = 33.0 Hz), 126.5, 126.4 (q, *J* = 3.6 Hz), 125.7, 124.1 (q, *J* = 273.1 Hz), 117.7, 114.0. ^{19}F NMR (376 MHz, $CDCl_3$) δ -62.48. ESI-MS (+), m/z: 269 $[M+H]^+$. EI-MS, m/z (%): 268 (100), 249 (10), 184 (12), 134 (10). Elemental analysis $C_{12}H_7F_3N_2S$ (268.03): calcd. C, 53.73; H, 2.63; N, 10.44; S, 11.95; found C, 53.91; H, 2.61; N, 10.48; S, 12.00.

5-(4-(methylsulfonyl)phenyl)imidazo[2,1-*b*]thiazole (3c)

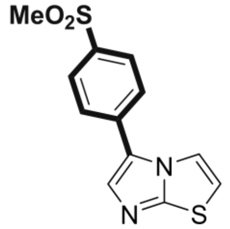

The crude was purified by flash chromatography on silica gel using a mixture of toluene/EtOAc (10:90) as eluent to give **3c** (70 mg, 50% yield) as a beige solid. M.p.=166-168 °C. ^{1}H NMR (500 MHz, DMSO-d_6) δ: 8.32 (d, J = 1.1 Hz, 1H), 8.00-7.93 (m, 4H), 7.82 (d, *J* = 4.5 Hz, 1H), 7.5 (dd, *J* = 4.5 Hz, *J* = 1.1 Hz, 1H), 3.26 (s, 3H). ^{13}C NMR (125 MHz, DMSO-d_6) δ: 151.7, 138.6, 134.6, 134.1, 127.9, 125.7, 125.1, 119.6, 114.9, 43.6. ESI-MS (+), m/z: 279 $[M+H]^+$.EI-MS, m/z (%): 278 (100), 215 (25), 199(30), 172 (10). Elemental analysis $C_{12}H_{10}N_2O_2S_2$ (278.02): calcd. C, 51.78; H, 3.62; N, 10.06; S, 23.04; found C, 51.65; H, 3.61; N, 10.10; S, 22.95.

Ethyl 4-(imidazo[2,1-*b*]thiazole-5-yl)benzoate (3d)

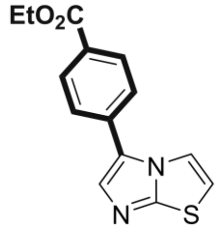

The crude was purified by flash chromatography on silica gel using a mixture of toluene/EtOAc (30:70) as eluent to give **3d** (47 mg, 34% yield) as a yellow solid. M.p.=150-152 °C. ^{1}H NMR (400 MHz, $CDCl_3$) δ: 8.14 – 8.12 (m, 2H), 7.72 (d, *J* = 4.58 Hz, 1H), 7.60 – 7.58 (m, 2H), 7.56 (d, *J* = 1.15 Hz, 1H), 6.96 (dd, *J* = 4.58 Hz, J = 1.15 Hz, 1H), 4.40 (q, *J* = 7.1, 2H), 1.42 (t, *J* = 7.1, 3H.) ^{13}C NMR (100 MHz, $CDCl_3$) δ 166.6, 152.1, 134.5, 134.2, 131.0, 129.6, 127.3, 125.4, 118.3, 114.2, 61.6, 14.8. ESI-MS (+), m/z: 273 $[M+H]^+$. EI-MS, m/z (%): 272 (100), 244 (80), 227 (40), 199 (20), 172 (7), 114 (7). Elemental analysis $C_{14}H_{12}N_2O_2S$ (272.06): calcd. C, 61.75; H, 4.44; N, 10.29; S, 11.77; found C, 61.60; H, 4.42; N, 10.20; S, 11.80.

5-(3-nitrophenyl)imidazo[2,1-b]thiazole (3e)

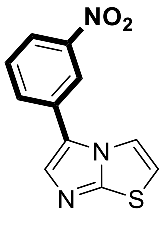

The crude was purified by flash chromatography on silica gel using a mixture of toluene/iPrOH (90:10) as eluent to give **3e** (49 mg, 40% yield) as a bright yellow solid. M.p.=124-125 °C. ^{1}H NMR (400 MHz, $CDCl_3$) δ: 8.38 – 8.37 (m, 1H), 8.20 – 8.17 (m,

1H), 7.87 – 7.83 (m, 1H), 7.73 (d, *J* = 4.54 Hz, 1H), 7.65 (t, *J* = 8.23 Hz, 1H), 7.60 (d, *J* = 1.13 Hz, 1H), 7.03 (dd, *J* = 4.54 Hz, J = 1.13 Hz, 1H) ^{13}C NMR (100 MHz, $CDCl_3$) δ 152.0, 149.0, 134.1, 131.7, 131.3, 130.5, 125.5, 122.1, 119.9, 117.6, 114.5. ESI-MS (+), m/z: 246 $[M+H]^+$. EI-MS, m/z (%): 245 (100), 199 (40), 172 (10), 141 (7), 75 (7). Elemental analysis $C_{14}H_{12}N_2O_2S$ (245.03): calcd. C, 53.87; H, 2.88; N, 17.13; S, 13.07; found C, 53.95; H, 2.90; N, 17.20; S, 13.10.

4-(imidazo[2,1-*b*]thiazol-5-yl)benzonitrile (3f)

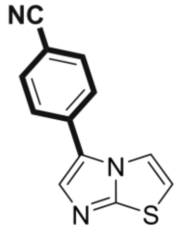

The crude was purified by flash chromatography on silica gel using a mixture of DCM/EtOAc (75:25) as eluent to give **3e** (40 mg, 35% yield) as an off-white solid. M.p.=219-221 °C. ^{1}H NMR (400 MHz, DMSO-*d*$_6$) δ: 8.33 (d, *J* = 4.4 Hz, 1H), 7.93 – 7.88 (m, 4H), 7.85 (d, *J* = 1.1 Hz, 1H), 7.48 (dd, *J* = 4.5 Hz, *J* = 1.1 Hz, 1H). ^{13}C NMR (100 MHz, DMSO-*d*$_6$) δ: 151.9, 134.8, 133.7, 133.1, 125.7, 125.1, 119.7, 118.9, 114.9, 109.0. ESI-MS (+), m/z: 226 $[M+H]^+$. EI-MS, m/z (%): 225 (100), 141 (15), 127(10), 114 (9). Elemental analysis $C_{12}H_7N_3S$ (225.04): calcd. C, 63.98; H, 3.13; N, 18.65; S, 14.23; found C, 64.15; H, 3.14; N, 18.70; S, 14.18.

5-(p-tolyl)imidazo[2,1-*b*]thiazole (3g)

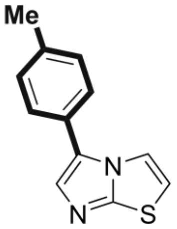

The crude was purified by flash chromatography on silica gel using a mixture of toluene/EtOAc (60:40) as eluent to give **3f** (10 mg, 9% yield) as a white solid. M.p.=115-117 °C. ^{1}H NMR (400 MHz, $CDCl_3$) δ: 7.62 (d, *J* = 4.5 Hz, 1H), 7.41 – 7.39 (m, 3H), 7.27 – 7.24 (m, 2H), 6.87 – 6.85 (m, 1H), 2.39 (s, 3H). ^{13}C NMR (100 MHz, $CDCl_3$) δ: 150.2, 137.7, 132.1, 130.0, 127.8, 127.2, 126.0, 117.8, 113.1, 21.4. ESI-MS (+), m/z: 215 $[M+H]^+$. EI-MS, m/z (%): 214 (100), 130 (20), 115(18), 103(9). Elemental analysis $C_{12}H_{10}N_2S$ (214.06): calcd. C, 67.26; H, 4.70; N, 13.07; S, 14.96; found C, 67.43; H, 4.69; N, 13.11; S, 15.00.

5-(pyridin-3-yl)imidazo[2,1-*b*]thiazole (3h)

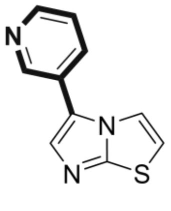

The crude was purified by flash chromatography on silica gel using a mixture of DCM/MeOH (95:5) as eluent to give **3g** (32 mg, 32% yield) as a yellow solid. M.p.=136-138 °C. ^{1}H NMR (400 MHz, $CDCl_3$) δ: 8.84 (d, *J* = 1.8 Hz, 1H), 8.6 (dd, J = 4.8, 1.6 Hz, 1H), 7.83 (dt, *J* = 8.1, 1.9 Hz, 1H), 7.68 (d, *J* = 4.5 Hz, 1H), 7.53 (d, *J* = 0.8 Hz, 1H), 7.41 (ddd, *J* = 8.0, 4.8, 0.9 Hz, 1H), 6.98 (dd, *J* = 4.5 Hz, *J* = 0.8 Hz, 1H). ^{13}C NMR (100 MHz, $CDCl_3$) δ: 151.6, 148.8, 146.8, 133.5, 133.1, 126.3, 124.4, 124.1, 117.5, 114.1. ESI-MS (+), m/z: 202 $[M+H]^+$. EI-MS,

m/z (%): 201 (100), 174 (10), 117(10), 76(9). Elemental analysis $C_{10}H_7N_3S$ (201.04): calcd. C, 59.68; H, 3.51; N, 20.88; S, 15.93; found C,59.88, H, 3.50, N, 20.81; S, 15.97.

Screening of the reaction conditions for the direct C2/C3 arylation of 1 with 4-bromotoluene (2f) or 4-iodotoluene (4b)

A mixture of **1** (63 mg, 0.5 mmol), CuI (190 mg, 1mmol), 4-bromotoluene (**2f**) or 4-iodotoluene (**4b**) (0.75 or 2 mmol), base (1 mmol) in DMA (2 mL) was stirred under argon for 48 or 72h at the selected temperature. After cooling the reaction mixture to room temperature, sodium sulphide nonahydrate (Na_2S-$9H_2O$, 1.5g, 3 mmol, 6 equiv.) was added to the crude mixture. DCM (approx. 20 mL) was added and the mixture was stirred for 30 minutes at room temperature then diluted with AcOEt (20 mL) and stirred again for another 30 minutes. The resulting mixture was filtered over Celite® and washed with AcOEt (15 mL) and DCM (15 mL). Biphenyl was added as internal standard, and the resulting solution was analysed by GLC-FID. Table 2 summarizes the results of this screening.

2-(p-tolyl)imidazo[2,1-*b*]thiazole (5a), 3-(p-tolyl)imidazo[2,1-*b*]thiazole (6a), 2,5-di-p-tolylimidazo[2,1-*b*]thiazole (8a) (entry 2, Table 2)

GLC-FID and UPLC-MS-DAD analyses of the crude reaction mixture obtained by the reaction of **1** with 4-bromotoluene (**2f**) (entry 1, Table 2) showed that it contains the C2 and C3 monoarylated imidazothiazoles **5a** and **6a**, respectively, and the C2,3 and C2,5 diarylated imidazothiazoles **7a** and **8a**. The purification of this complex reaction mixture by flash chromatography using toluene/EtOAc (70:30) as eluant on silica gel allowed us to isolate compounds **5a**, **6a**, and **8a** in sufficient amounts to confirm their structure. The attribution of NMR chemical shifts was based according to literature data.[1]

2-(p-tolyl)imidazo[2,1-*b*]thiazole (5a)

The ^{1}H-NMR spectrum of this compound (13.5 mg, 13 % yield, NMR purity of 90%) shows a singlet at 7.58 ppm attributable at the H3 proton of the imidazole core. The signals of the protons H5 and H6 can be observed at 7.42 and 7.30 ppm, respectively, with $J_{5,6}$ ~ 1.4 Hz. The signal relative to the H2 proton is not present as well as the coupling H3-H2.

^{1}H NMR (400 MHz, Chloroform-*d*) δ 7.58 (s, 1H), 7.42 (d, *J* = 1.38 Hz, 1H), 7.41 – 7.37 (m, 2H), 7.30 (d, *J* = 1.35 Hz, 1H), 7.25 – 7.20 (m, 2H), 2.38 (s, 3H).

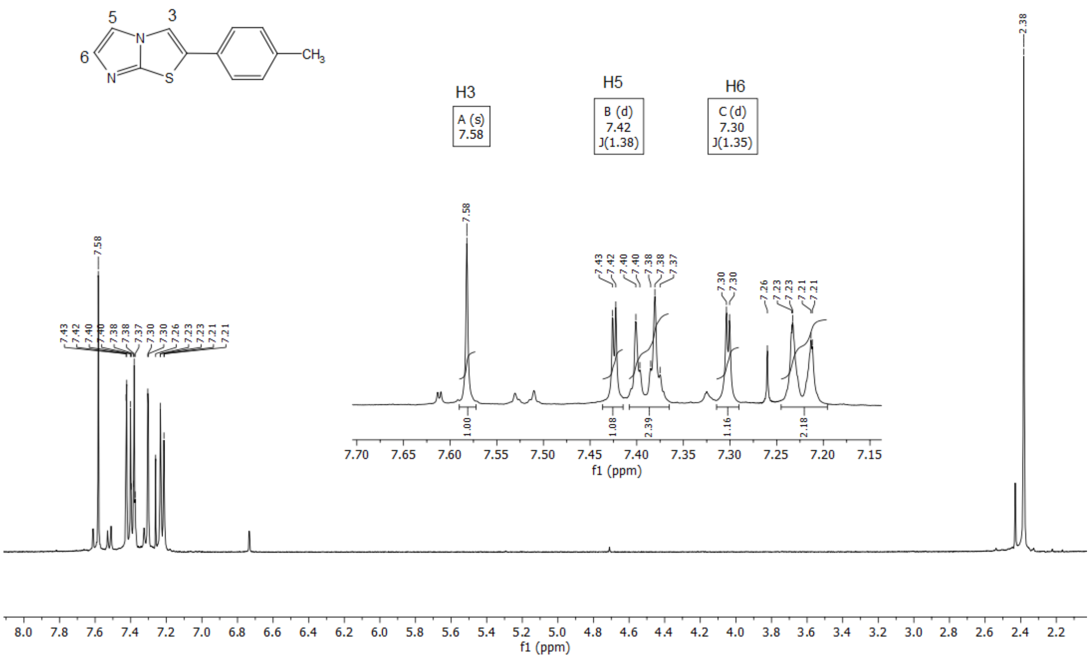

3-(p-tolyl)imidazo[2,1-*b*]thiazole (6a)

The ^{1}H-NMR spectrum of this compound (11.8 mg, 11 % yield, NMR purity of 95%) shows a doublet at 7.61 ppm with $J_{5,6}$ = 1.57 Hz, attributable to the H5 proton of the imidazole core and a doublet at 6.73 ppm with $J_{2,6}$ = 1.08 Hz attributable to the H2 proton. Indeed, the proton H6 resonates at 7.38 as triplet with J = 1.37 Hz. The signal relative to the H3 proton is not present as well as the coupling H2-H3.

^{1}H NMR (400 MHz, Chloroform-*d*) δ 7.61 (d, *J* = 1.57 Hz, 1H), 7.54 – 7.48 (m, 2H), 7.37 (t, *J* = 1.37 Hz, 1H), 7.34 – 7.28 (m, 2H), 6.73 (d, *J* = 1.08 Hz, 1H), 2.43 (s, 3H).

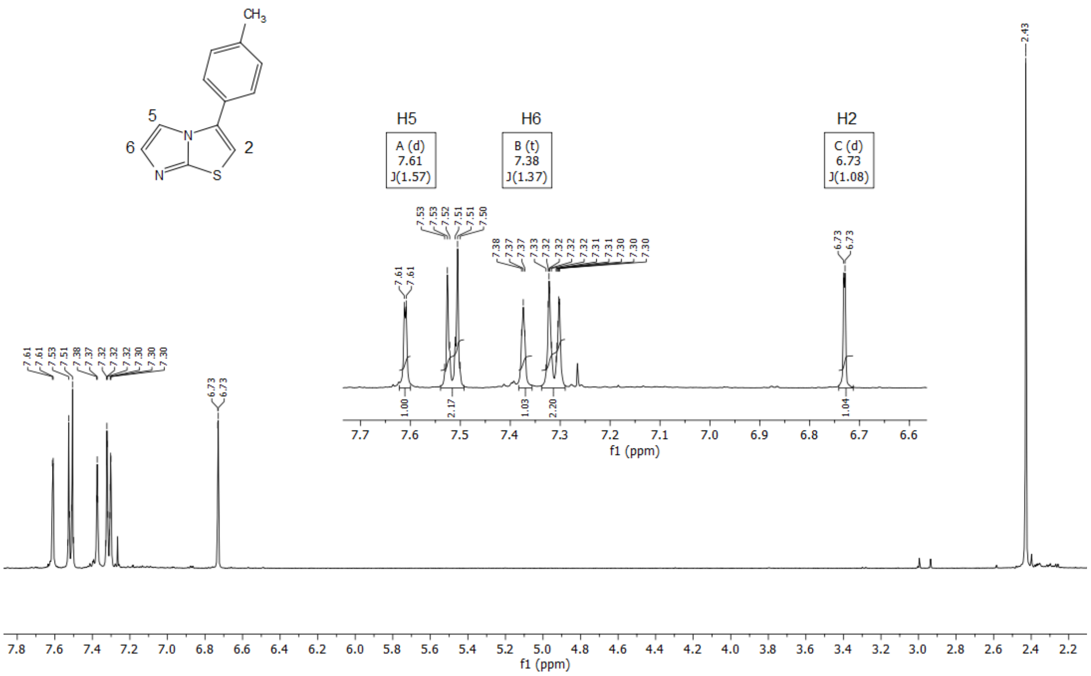

2,5-di-p-tolylimidazo[2,1-*b*]thiazole (8a)

The ^{1}H-NMR spectrum of this compound (15.2 mg, 10 % yield, NMR purity of 88%) shows two singlets at 7.77 and 7.36 ppm attributable to the H3 and H6 protons of the imidazole core, respectively, lacking of the peculiar coupling $J_{2,3}$, $J_{5,6}$ and $J_{2,6}$. The absence of signals in the usual chemical shift region of H5 and H2 confirms the 2,5-derivatization.

^{1}H NMR (401 MHz, Chloroform-*d*) δ 7.77 (s, 1H), 7.48 – 7.39 (m, 4H), 7.36 (s, 1H), 7.33 – 7.17 (m, 5H), 2.41 (s, 3H), 2.38 (s, 3H).

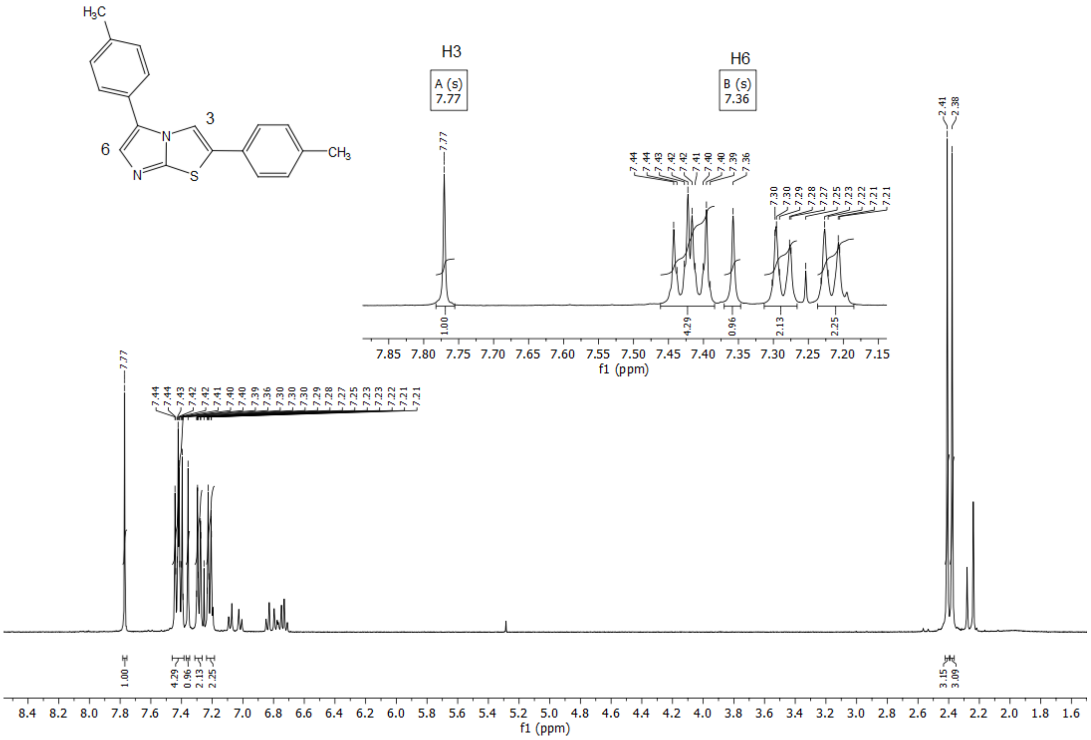

2,3-bis(4-methylphenyl)imidazo[2,1-*b*]thiazole (7a)

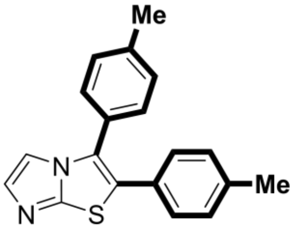

The crude reaction product obtained by the reaction of **1** with 4-bromotoluene (**2f**), (entry 3, Table 2) was purified by flash chromatography by using a mixture of petroleum ether/EtOAc (70:30) as eluent to give **7a** (68% yield, 103 mg) as a white crystalline solid. M.p.= 208-209 °C. ^{1}H NMR (400 MHz, $CDCl_3$): 7.36 – 7.29 (m, 4H), 7.23 (d, *J* = 7.9 Hz, 2H), 7.16 (d, *J* = 8.2 Hz, 2H), 7.07 (d, *J* = 8.0 Hz, 2H), 2.43 (s, 3H), 2.35 (s, 3H). ^{13}C NMR (100 MHz, $CDCl_3$) δ: 147.2, 139.5, 138.3, 133.6, 129.9, 129.4, 129.2, 128.9, 126.8, 126.6, 125.7, 111.6, 21.4, 21.2. ESI-MS m/z 305 $[M+H]^+$. EI-MS m/z (%) 305 (23), 304 (100), 303 (45), 206 (10). Elemental analysis $C_{19}H_{16}N_2O_2S$ (336.09): calcd. C, 67.84; H, 4.79; N, 8.33; S, 9.53; found C, 68.01; H,4.80; N, 8.35; S, 9.56. This compound was also obtained in isolated yields of 51%, 55%, 85%, and 62% as reported in Table 2, entries 4-7.

Table S1. Base screening for the synthesis of 2,3-di-*p*-tolylimidazothiazole **7a**

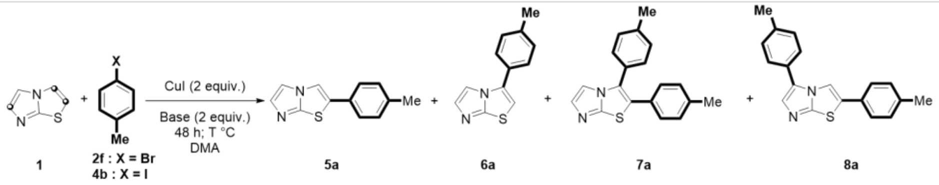

Entry[a]	X (equiv)	Base	Conversion of **1**[b]	**5a**/**6a** AP% ratio[c]	**7a**/**8a** AP% ratio[c]	Mono/diarylated product ratio (AP%)	Yield of **7a**[d]
1	Br (1.5)	LiO*t*-Bu	90	55/45	>99/<1	75/25	<5
2	Br (1.5)	KO*t*-Bu	92	49/51	>99/<1	61/39	<5
3	Br (1.5)	K_2CO_3	67	59/41	93/7	85/14	18
4	Br (1.5)	K_3PO_4	57	61/39	89/11	84/16	10
5	Br (1.5)	KOAc	37	55/45	68/32	94/6	6
6	Br (4.0)	K_2CO_3	95	55/45	82/18	38/62	49
7	I (4.0)	Cs_2CO_3	98	58/42	91/9	9/91	60

[a] General conditions: **1** (0.5 mmol), **2f** or **4b** (1.5 or 4.0 equiv, see table), base (2.0 equiv), CuI (2.0 equiv), DMA (2 mL), 140 °C, 48 hours, unless otherwise reported. [b] Determined by GLC-FID analysis, using biphenyl as internal standard. [c] The **5a**/**6a** ratio (i.e. C2/C3) and the **7a**/**8a** (i.e. C2,3/C2,5) ratio were expressed as the ratio between the area percent (AP%) of the compounds in the UPLC-MS-DAD chromatogram. AP% values are uncorrected for the differences in response factors. [d] Determined by GLC-FID analysis using biphenyl as internal standard.

General scope for C-2 and C-3 diarylation of imidazo[2,1-*b*]thiazoles (7a-g)

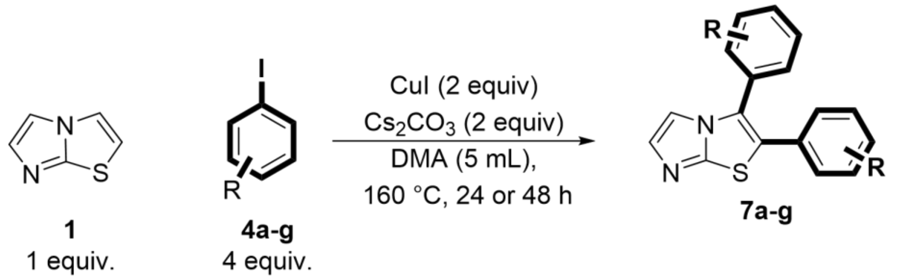

In a 25-mL two-neck flask previously dried, flushed and maintained under argon atmosphere, equipped with a condenser and magnetic stirrer, all the solid reagents were introduced: **1** (126 mg, 1 mmol), Cs_2CO_3 (650 mg, 2 mmol) and CuI (380 mg, 2 mmol). Two more argon-*vacuum* cycles were made prior addition of DMA (5 mL) and aryl-iodide **4a-g** (4 mmol). The reaction was held at 160 °C for 24 or 48 hours (Scheme 2). After bringing the reaction mixture to room temperature, sodium sulfide nonahydrate (Na_2S-$9H_2O$, 1.5g, 6 mmol, 6 equiv.) was added to the crude mixture. DCM (approx. 20 mL) was added and the mixture was stirred for 30 minutes at room temperature then diluted with AcOEt (20 mL) and stirred again for another 30 minutes. The resulting mixture was filtered over Celite® and washed with AcOEt (15 mL) and DCM (15 mL). The resulting solution was concentrated under reduced pressure and analysed (GC-FID, GC-MS and UPLC-MS). The residue was purified by flash chromatography on silica gel.

2,3-bis(4-methoxyphenyl)imidazo[2,1-*b*]thiazole (7b)

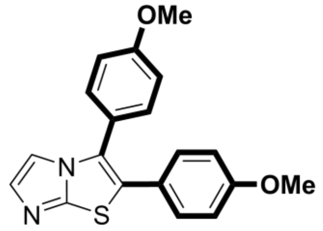

The crude was purified by flash chromatography by using a mixture of DCM/EtOAc (85:15) as eluent to give **7b** (71% yield, 234 mg) as a dusty yellow solid. M.p.= 136-138°C. ^{1}H NMR (400 MHz, DMSO-d_6) δ: 7.44 – 7.37 (m, 2H), 7.24 – 7.19 (m, 2H), 7.09 – 7.02 (m, 3H), 6.95 – 6.89 (m, 3H), 3.81 (s, 3H), 3.75 (s, 3H). ^{13}C NMR (100 MHz, DMSO-d_6) δ: 160.0, 159.4, 130.9, 130.2, 123.8, 123.3, 121.2, 114.8, 114.4, 55.8, 55.2. ESI-MS m/z 337 $[M+H]^+$. EI-MS m/z (%) 337.00 (31), 336.10 (100), 336.00 (71), 321.05 (30), 223.05 (17). Elemental analysis $C_{19}H_{16}N_2O_2S$ (336.09): calcd. C, 67.84; H, 4.79; N, 8.33; S, 9.53; found C, 68.09; H,4.80; N, 8.58; S, 9.57.

2,3-bis(4-nitrophenyl)imidazo[2,1-*b*]thiazole (7c)

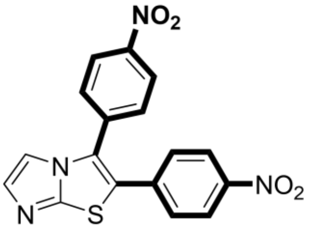

The crude was purified by flash chromatography by using a mixture of DCM/EtOAc (80:10) as eluent to give **7c** (75% yield, 275 mg) as an orange solid. M.p.= 227-230°C. ^{1}H NMR (400 MHz, DMSO-d_6) δ: 8.40 – 8.31 (m, 2H), 8.25 – 8.18 (m, 2H), 7.87 – 7.82 (m, 2H), 7.72 (d, J = 1.6 Hz, 1H), 7.60 – 7.54 (m, 2H), 7.40 (d, J = 1.6 Hz, 1H). ^{13}C NMR (100 MHz, DMSO-d_6) δ: 148.1, 147.2, 145.9, 137.4,

134.7, 131.2, 130.3, 127.1, 124.7, 124.5, 124.3, 113.0. ESI-MS m/z 367 [M+H]$^+$. EI-MS m/z (%) 367.10 (22), 366.15 (100), 336.10 (16), 319.10 (19), 272.25 (15). Elemental analysis $C_{17}H_{10}N_4O_4S$ (366.04): calcd. C, 55.74; H, 2.75; N, 15.29; S, 8.75; found C,55.90; H,2.74; N, 15.34; S, 8.77.

2,3-bis(4-chlorophenyl)imidazo[2,1-*b*]thiazole (7d)

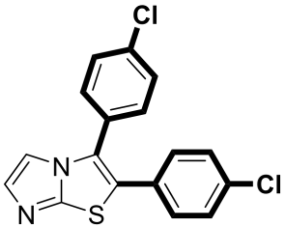

The crude was purified by flash chromatography by using a mixture of DCM/EtOAc (93:7) as eluent to give **7d** (73% yield, 252 mg) as a yellow solid. M.p.= 155-157°C. ^{1}H NMR (400 MHz, $CDCl_3$) elaborate upon addition of TMS as reference δ: 7.45 – 7.40 (m, 2H), 7.38 – 7.34 (m, 2H), 7.31 (s, 2H), 7.27 – 7.23 (m, 2H), 7.19 – 7.15 (m, 2H). ^{13}C NMR (100 MHz, $CDCl_3$) δ: 146.9, 135.8, 134.7, 134.1, 130.6, 130.6, 130.3, 130.3, 130.3, 129.8, 129.8, 129.2, 129.1, 127.7, 126.1, 125.2, 111.5. ESI-MS m/z 346 [M+H]$^+$. EI-MS m/z (%) 346.00 (69), 345.10 (23), 345.00 (29), 344.05 (100), 343.05 (49), 246.05 (25). Elemental analysis $C_{17}H_{10}$ Cl_2N_2S (343,99): calcd. C, 59.14; H, 2.92; N, 8.11; S, 9.29; found C, 59.30; H,2.91; N, 8.18; S, 9.31.

Diethyl 4,4'-(imidazo[2,1-*b*]thiazole-2,3)dibenzoate (7e)

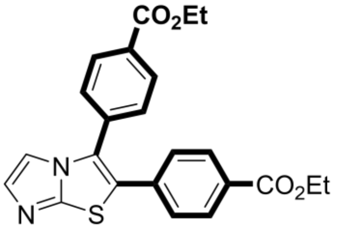

The crude was purified by flash chromatography by using a mixture of DCM/EtOAc (85:15) as eluent to give **7e** (25% yield, 105 mg) as a yellow oil. ^{1}H NMR (400 MHz, $CDCl_3$) δ: 8.17 – 8.10 (m, 2H), 7.99 – 7.92 (m, 2H), 7.58 – 7.50 (m, 2H), 7.40 – 7.28 (m, 4H), 4.47 – 4.26 (m, 4H), 1.40 (dt, *J* = 15.4, 7.1 Hz, 6H). ^{13}C NMR (100 MHz, $CDCl_3$) δ: 165.6, 165.5, 147.2, 135.6, 134.3, 133.5, 131.7, 130.5, 130.4, 129.9, 129.3, 128.9, 126.9, 125.9, 111.6, 61.3, 61.1, 14.2, 14.2. ESI-MS m/z 421 [M+H]$^+$. EI-MS m/z (%) 421.20 (28), 420.20 (100), 375.20 (13), 363.20 (15). Elemental analysis $C_{23}H_{20}N_2O_4S$ (420.11): calcd. C, 65.70; H, 4.79; N, 6.66; S, 7.62; found C,65.43; H,4.8; N, 6.64; S, 7.64.

2,3-bis((1-naphthalen)imidazo[2,1-*b*]thiazole (7f)

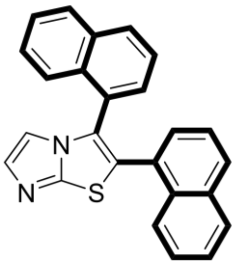

The crude was purified by flash chromatography by using a mixture of DCM/EtOAc (95:5) as eluent to give **7f** (80% yield, 252 mg) as a light brown solid. M.p.= 202-204°C. ^{1}H NMR (400 MHz, $CDCl_3$) elaborate upon addition of TMS as reference δ 8.16 – 8.08 (m, 1H), 7.86 – 7.67 (m, 5H), 7.52 – 7.26 (m, 7H), 7.25 – 7.18 (m, 2H), 6.92 (s, 1H). ^{13}C NMR (100 MHz, CDCl3) δ 148.1, 133.8, 133.7, 133.4, 132.5, 131.2, 130.2, 129.8, 129.7, 129.3, 128.7, 128.4, 128.3, 128.3, 127.0, 126.7, 126.4, 126.2, 125.6,125.3, 125.3, 124.9, 124.9, 112.5. ESI-MS m/z 377 [M+H]$^+$. EI-MS m/z (%) 377.20 (29), 376.15 (100), 375.15 (57), 377.20 (16), 276.15 (17). Elemental analysis $C_{25}H_{16}N_2S$ (376.10): calcd. C, 79.76; H, 4.28; N, 7.44; S, 8.52; found C, 80.01; H,4.29; N, 7.46; S, 8.54.

2,3-di(3-pyridin)imidazo[2,1-*b*]thiazole (7g)

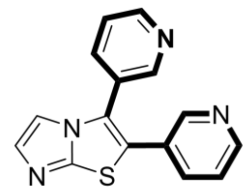

The crude was purified by flash chromatography by using a mixture of DCM/MeOH (93:7) as eluent to give **7g** (75% yield, 195 mg) as a pale pink solid. M.p.= 172-174 °C. ^{1}H NMR (400 MHz, $CDCl_3$) δ: 8.75 – 8.68 (m, 2H), 8.56 (dd, *J* = 4.9, 1.6 Hz, 1H), 8.52 (dd, *J* = 2.3, 0.9 Hz, 1H), 7.81 (ddd, *J* = 7.9, 2.3, 1.6 Hz, 1H), 7.61 (ddd, *J* = 7.9, 2.3, 1.6 Hz, 1H), 7.45 (ddd, *J* = 7.9, 4.9, 0.9 Hz, 1H), 7.38 (s, 2H), 7.29 (ddd, *J* = 7.9, 4.9, 0.9 Hz, 1H). ^{13}C NMR (100 MHz, $CDCl_3$) δ: 150.9, 150.0, 149.8, 149.7, 147.2, 136.7, 136.4, 134.6, 127.4, 125.4, 125.0, 124.1, 123.9, 123.7, 111.5. ESI-MS m/z 279 $[M+H]^+$. EI-MS m/z (%) 279.10 (21), 278.10 (100), 277.10 (80), 179.10 (10). $C_{15}H_{10}N_4S$ (278.06): calcd. C, 64.73; H, 3.62; N, 20.13; S, 11.52; found C, 64.52; H,3.63; N, 20.18; S, 11.48.

DFT Calculations

Affinity towards electrophiles

Electrophile affinities and deprotonation energies were computed by optimizing the geometries of the reactants and products with analytical gradients using the B3LYP[2] exchange-correlation functional and the 6-311++G(2df,2pd)[3] basis set. All structures were confirmed to be *minima* by computing the vibrational frequencies using analytical second derivatives. The latter was also used to compute the thermal contributions to the absolute free energies of all molecules. Solvation effects were accounted with the Polarizable Continuum Model[4], using the SMD[5] parametrization of the cavity and non-electrostatic contributions. All the calculations were performed using the Gaussian 16 suite of programs.[6]

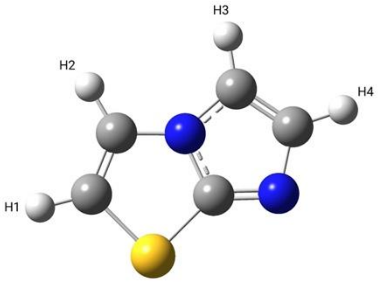

Theory level: B3LYP/6-311++G(2df,2pd) + SMD

Free energy of affinity to the electrophile toward electrophile at 298K

	**G(Br+)**	**G(IT)**	**G([IT-Br+])**	**ΔG(AffE) kcal/mol**	**DG(Prot) kcal/mol**
**H1**	-2573.700831	-700.695361	-3274.62798	145.4	26.03
**H2**	-2573.700831	-700.695361	-3274.619382	140.1	2571
**H3**	-2573.700831	-700.695361	-3274.653125	161.2	32.04
**H4**	-2573.700831	-700.695361	-3274.634502	149.5	41.81

Theory level: B3LYP/6-311++G(2df,2pd) + SM

Internal energy of affinity toward electrophile

	**E(Br+)**	**E(IT)**	**E([IT-Br+])**	**ΔE(AffE) kcal/mol**
**H1**	-2573.684655	-700.7497864	-3274.679797	154.0
**H2**	-2573.684655	-700.7497864	-3274.671481	148.7
**H3**	-2573.684655	-700.7497864	-3274.706182	170.5

**H4**	-2573.684655	-700.7497864	-3274.686517	158.2

Theory level: B3LYP/6-311++G(2df, 2pd) + SMD

Internal energy of affinity toward electrophile at 160 °C

	**G(Br+)**	**G(IT)**	**G([IT-Br+])**	**ΔG(AffE) kcal/mol**
**H1**	-2573.70944	-700.713114	-3274.64860	141.9
**H2**	-2573.70944	-700.713114	-3274.63986	136.4
**H3**	-2573.70944	-700.713114	-3274.67343	157.4
**H4**	-2573.70944	-700.713114	-3274.65496	145.8

Affinity towards electrophiles: imidazo[2,1-*b*]thiazole – Cu complex

Theory level: B3LYP/6-311++G(2df,2pd) + SMD

Internal energy of affinity toward electrophile

	**G(Br+)**	**G([IT-Br+])**	**ΔG(AffE) kcal/mol**	**DG(Prot) kcal/mol**
**H1**	-2573.700831	-4915.029178	138.1	26.03
**H2**	-2573.700831	-4915.022417	133.8	25.71
**H3**	-2573.700831	-4915.050083	151.2	32.04
**H4**	-2573.700831	-4915.031267	139.4	41.81

Theory level: B3LYP/6-311++G(2df,2pd) + SMD

Internal energy of affinity toward electrophile

	**E(Br+)**	**E(IT)**	**E([IT-Br+])**	**ΔE(AffE) kcal/mol**
**H1**	-2573.684655	-2341.140656	-4915.078428	158.8
**H2**	-2573.684655	-2341.140656	-4915.071065	154.2
**H3**	-2573.684655	-2341.140656	-4915.100323	172.6
**H4**	-2573.684655	-2341.140656	-4915.081318	160.6

Theory level: B3LYP/6-311++G(2df,2pd) + SMD

Internal energy of affinity toward electrophile

	**G(IT)**	**ΔG(AffE) kcal/mol**

**H1**	-2341.132896	117.2
**H2**	-2341.132896	113.0
**H3**	-2341.132896	130.4
**H4**	-2341.132896	118.6

Deprotonation

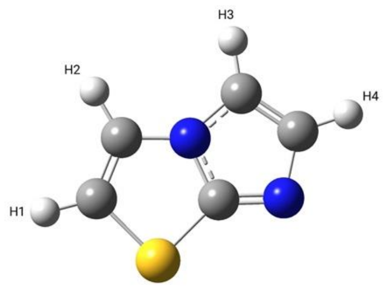

Theory level: B3LYP/6-311++G(2df,2pd) + SMD

Internal energy of affinity toward electrophile

	G(AcO-)	G(AcOH)	G([IT-H-])	ΔG(Prot) kcal/mol
**H1**	-228.686	-229.155	-700.185	26.03
**H2**	-228.686	-229.155	-700.185	25.71
**H3**	-228.686	-229.155	-700.175	32.04
**H4**	-228.686	-229.155	-700.160	41.81

Theory level: B3LYP/6-311++G(2df,2pd) + SMD

Internal energy of affinity toward electrophile

	G(AcO-)	G(AcOH)	G([IT-H-])	ΔG(Prot) kcal/mol
**H1**	-228.702	-229.169	-700.203	26.79
**H2**	-228.702	-229.169	-700.203	26.48
**H3**	-228.702	-229.169	-700.193	32.82
**H4**	-228.702	-229.169	-700.178	42.52

HOMO vs Potential

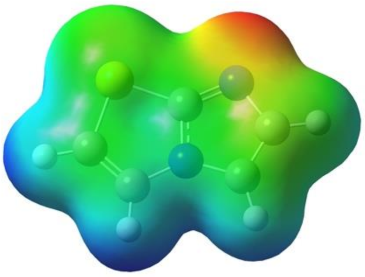

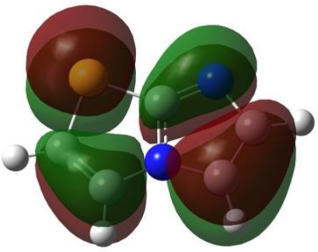

NMR spectra

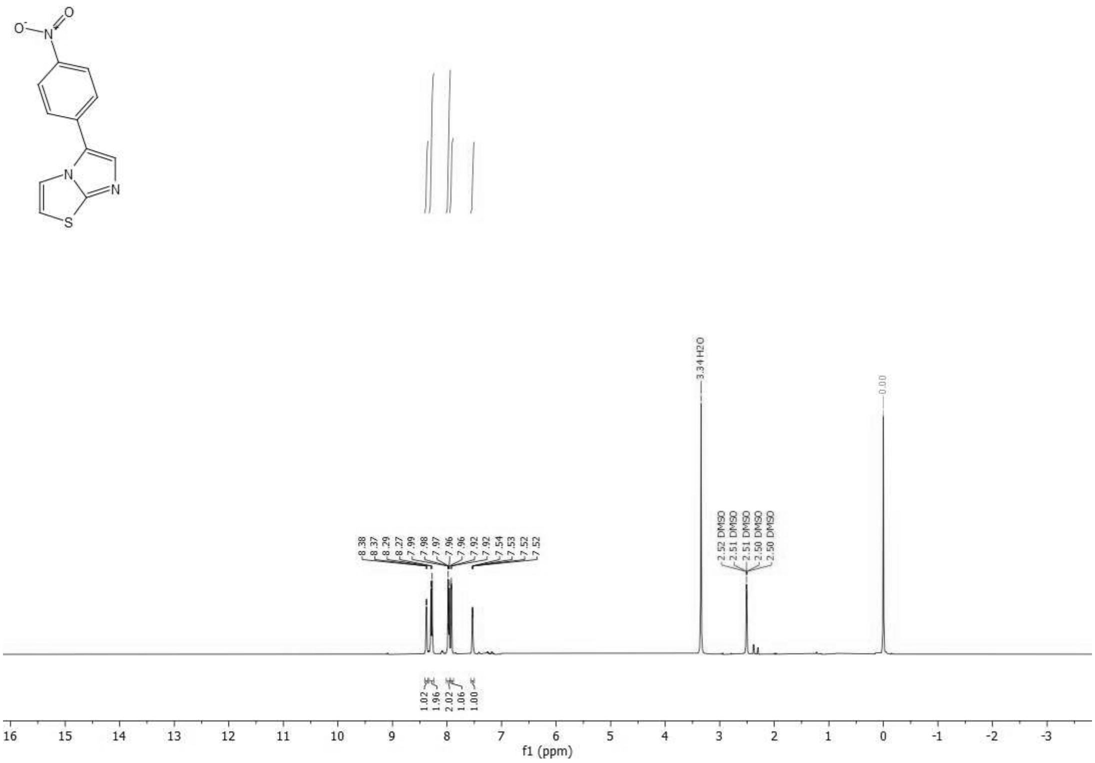

*Figure S1 5(4-nitrophenyl)imidazo[2,1-b]thiazole (**3a**) ^{1}H NMR*

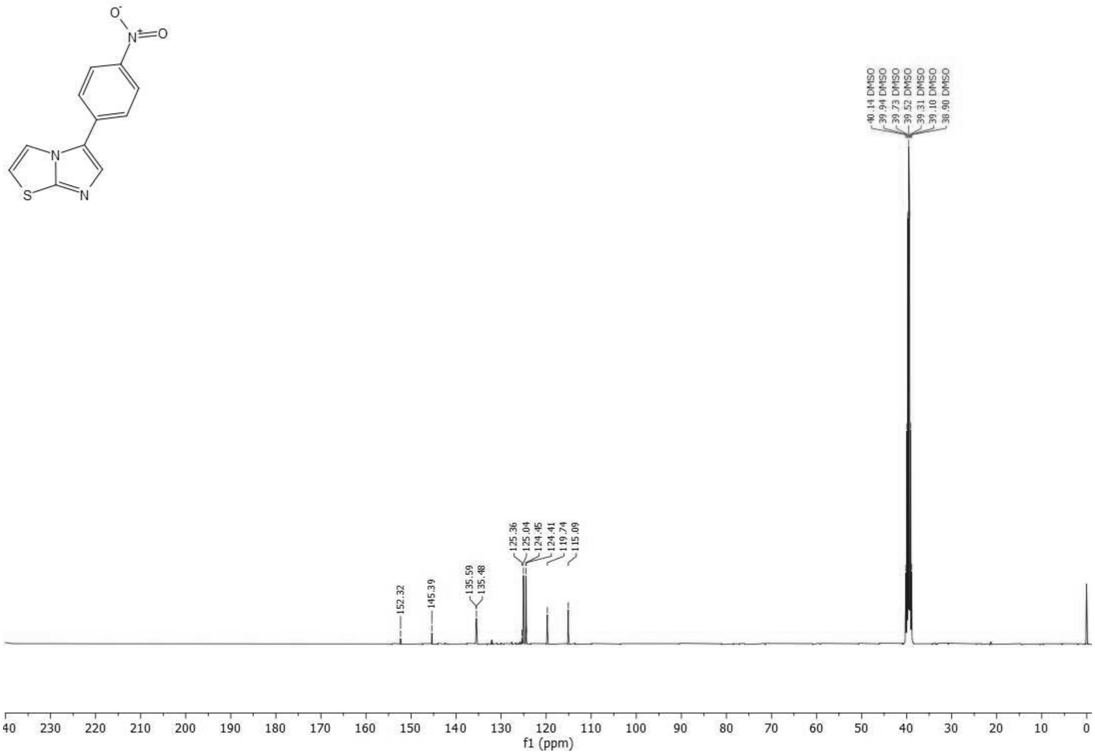

*Figure S1 5(4-nitrophenyl)imidazo[2,1-b]thiazole (**3a**) ^{13}C NMR*

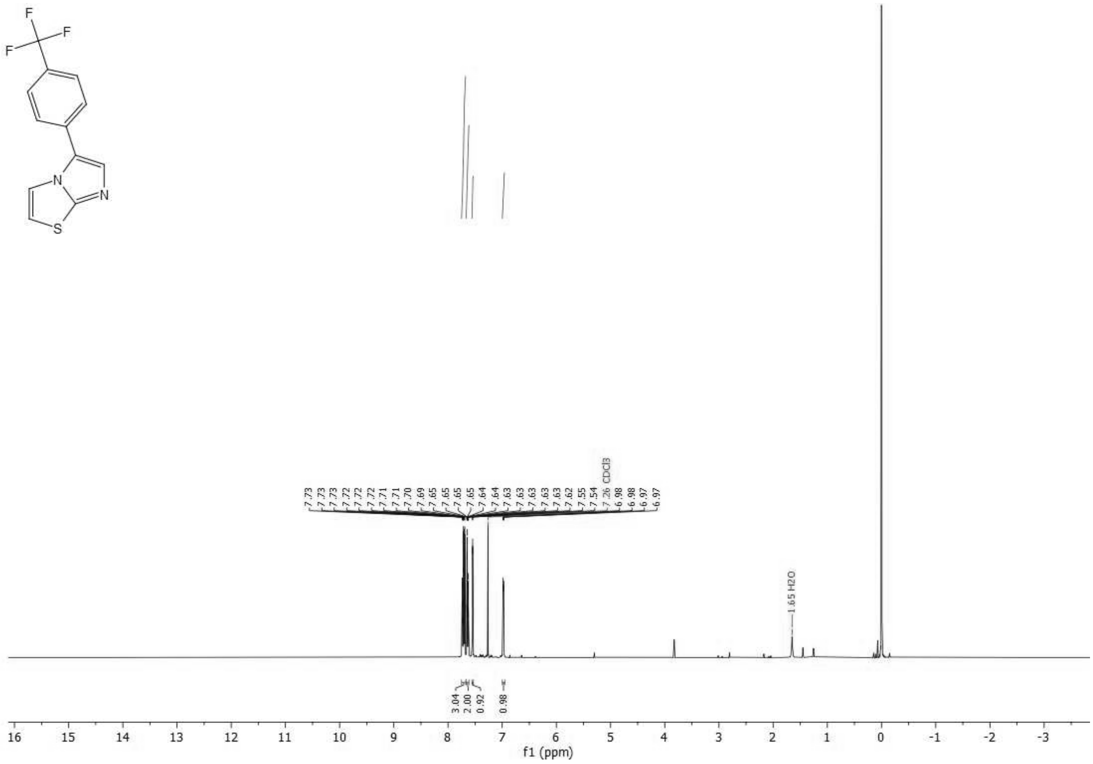

*Figure S2 5-(4-(trifluoromethyl)phenyl)imidazo[2,1-b]thiazole (**3b**) ^{1}H NMR*

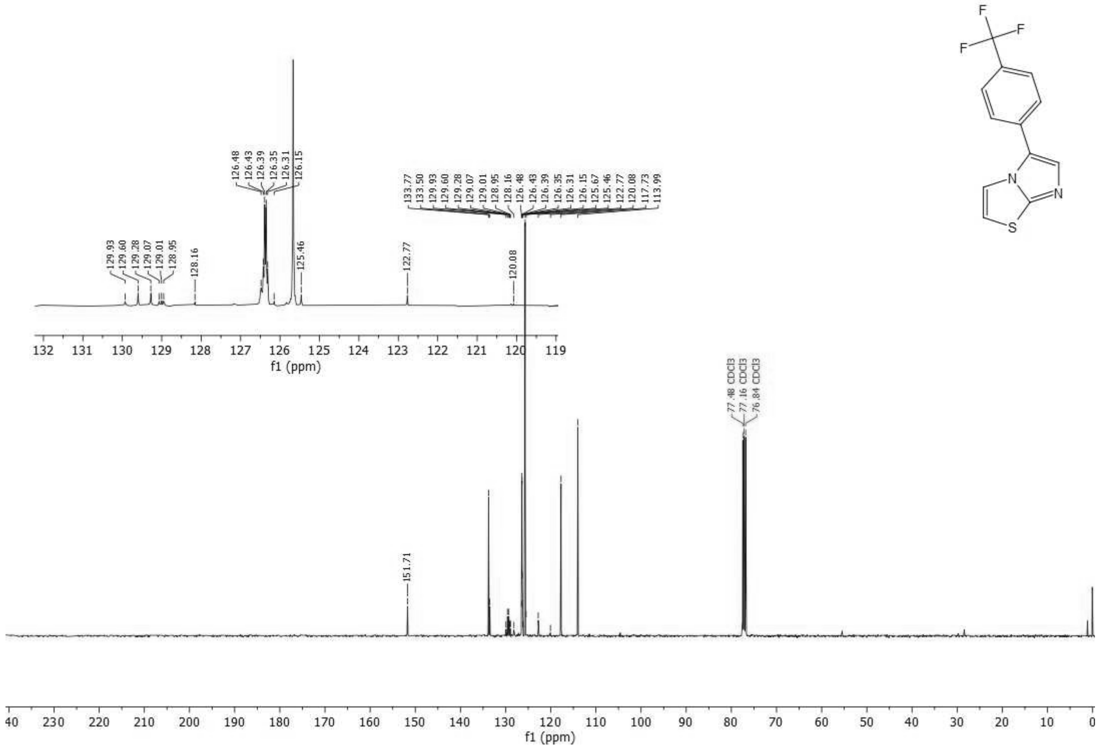

*Figure S3 5-(4-(trifluoromethyl)phenyl)imidazo[2,1-b]thiazole (**3b**) ^{13}C NMR*

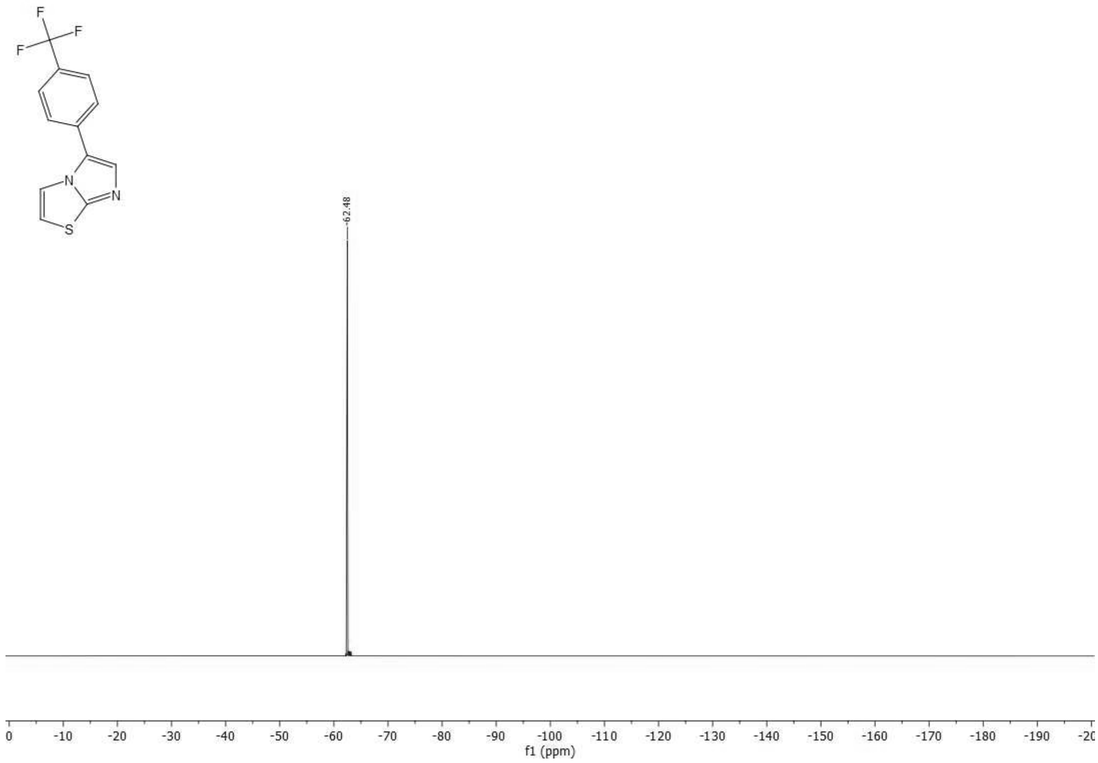

*Figure S4 5-(4-(trifluoromethyl)phenyl)imidazo[2,1-b]thiazole (**3b**) ^{19}F NMR*

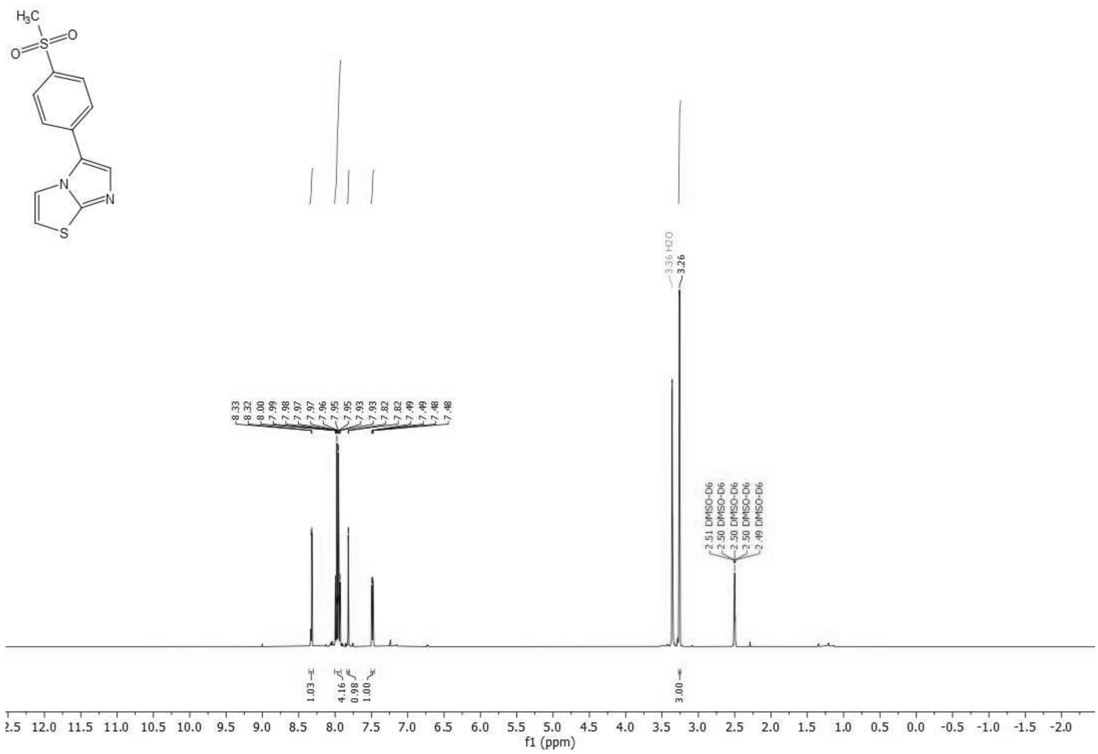

*Figure S5 5-(4-(methylsulfonyl)phenyl)imidazo[2,1-b]thiazole (**3c**) ^{1}H NMR*

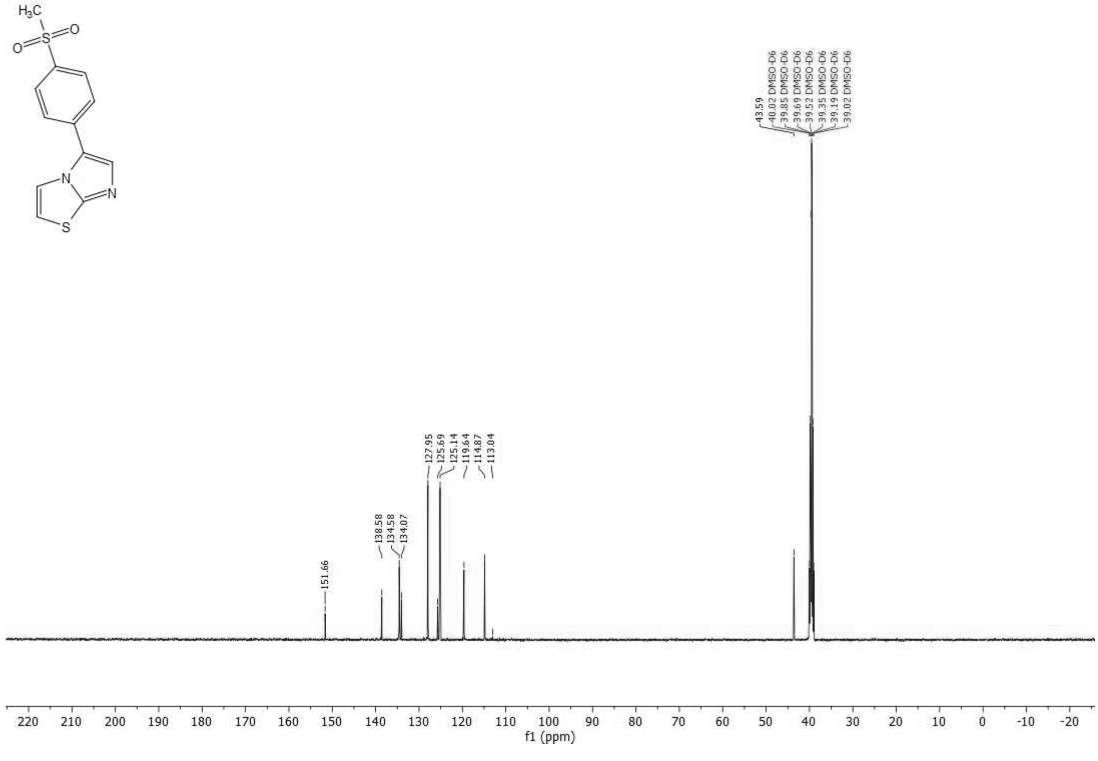

*Figure S6 5-(4-(methylsulfonyl)phenyl)imidazo[2,1-b]thiazole (**3c**) ^{13}C NMR*

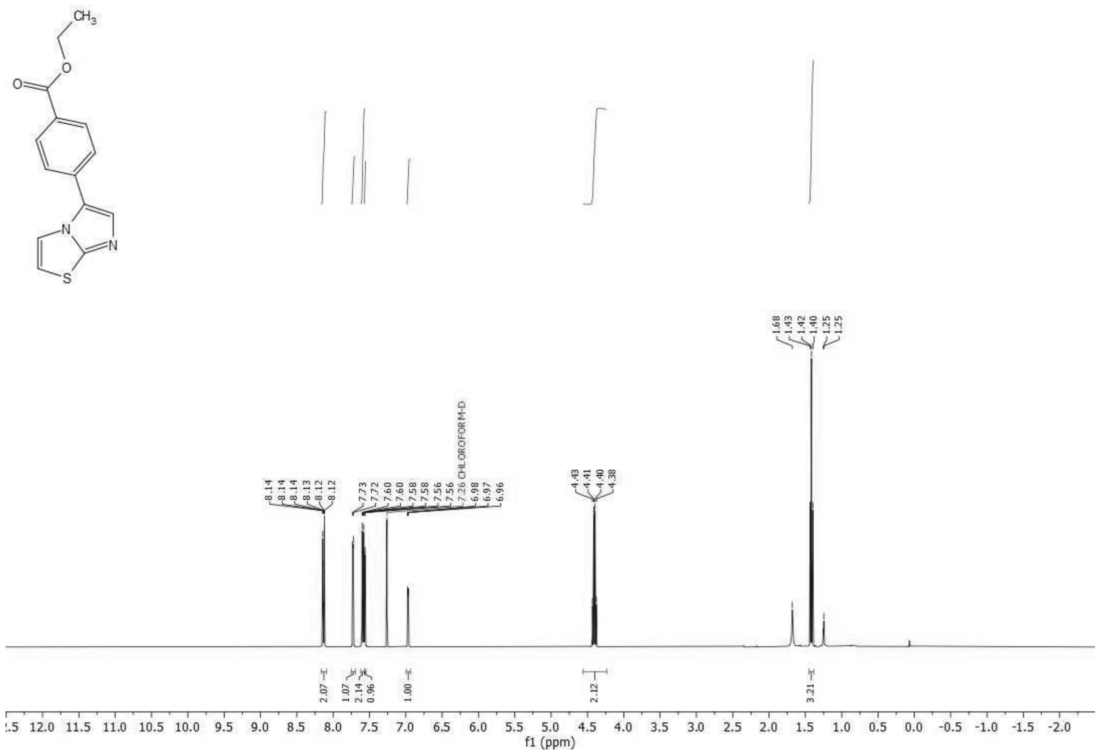

*Figure S7 Ethyl 4-(imidazo[2,1-b]thiazole-5-yl)benzoate) (**3d**) ^{1}H NMR*

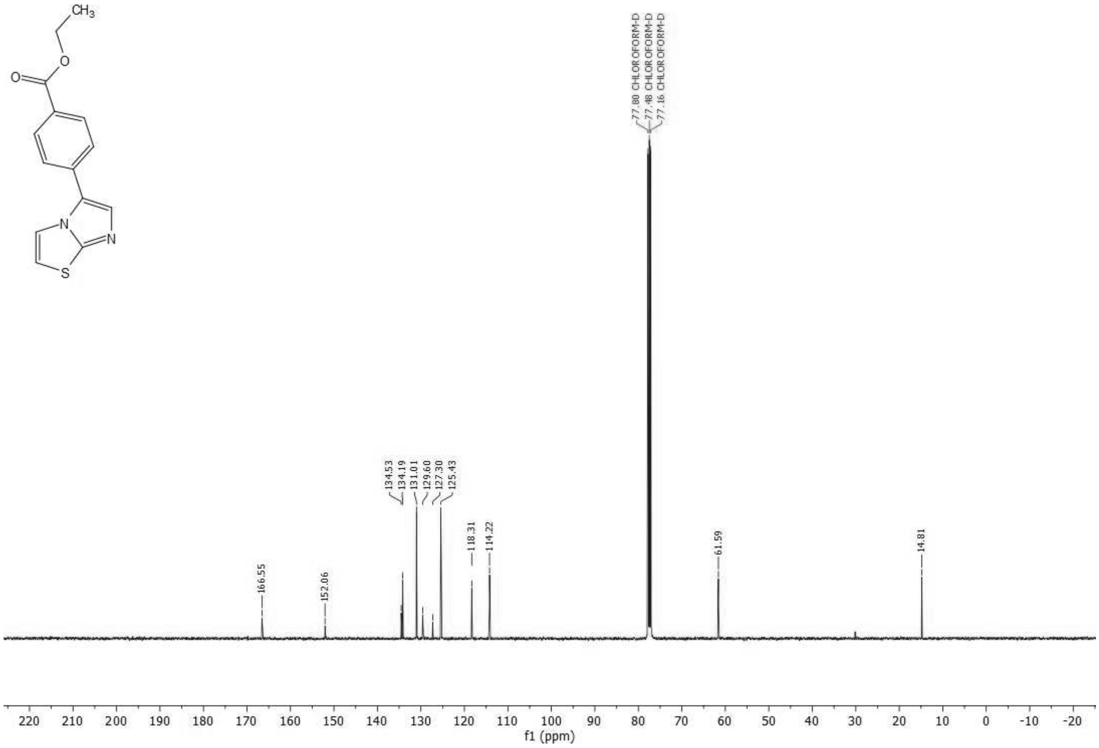

*Figure S8 Ethyl 4-(imidazo[2,1-b]thiazole-5-yl)benzoate) (**3d**) ^{13}C NMR*

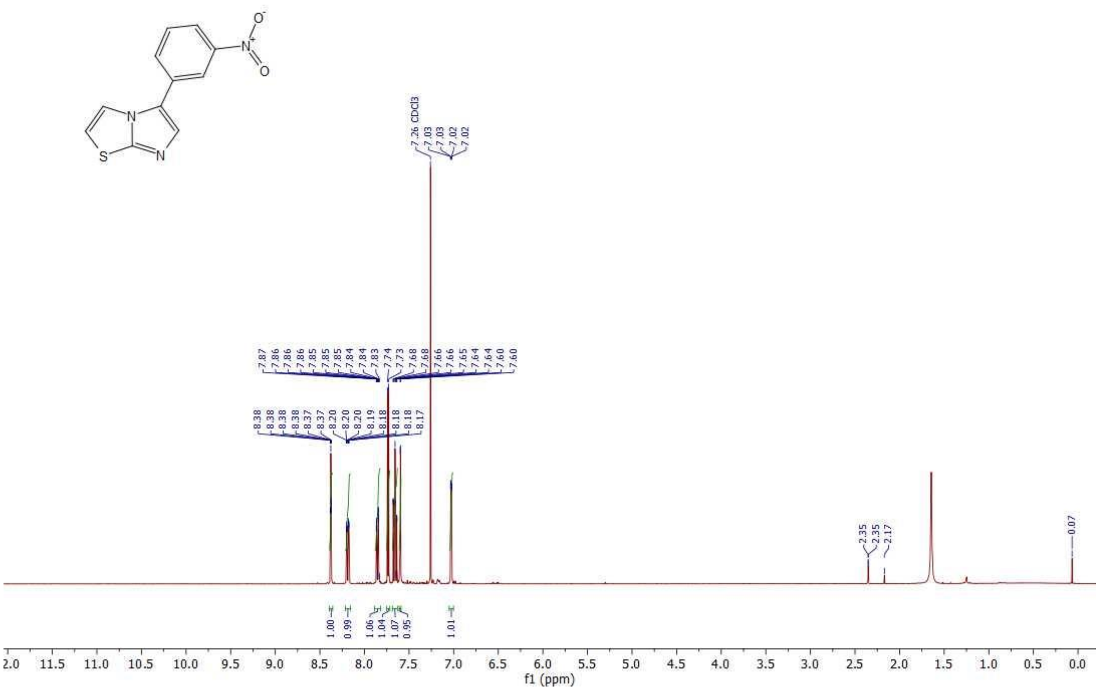

*Figure S10 5-(3-nitrophenyl)imidazo[2,1-b]thiazole (**3e**) ^{1}H NMR*

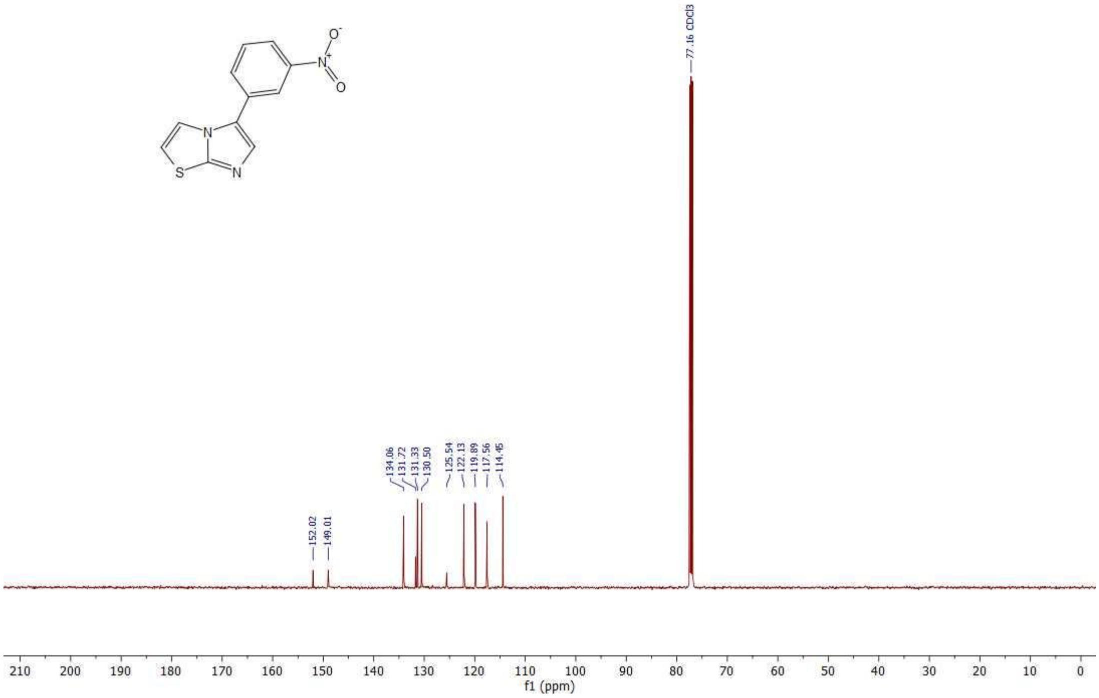

*Figure S11 5-(3-nitrophenyl)imidazo[2,1-b]thiazole (**3e**) ^{13}C NMR*

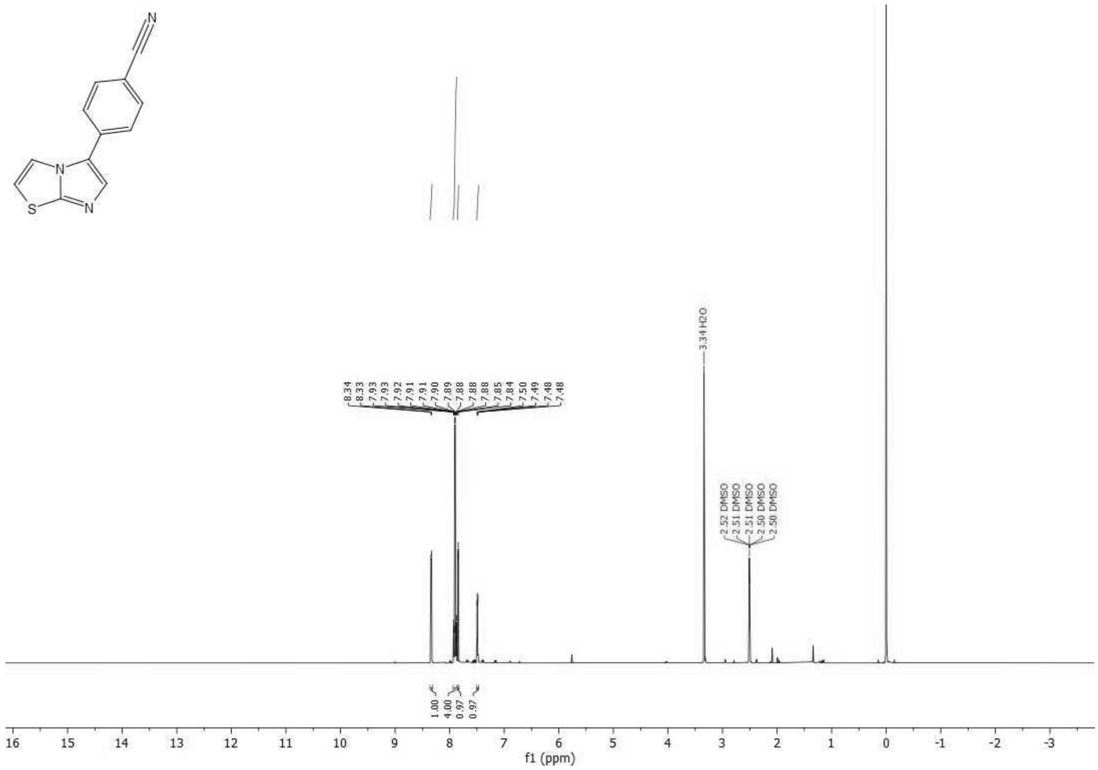

*Figure S9 4-(imidazo[2,1-b]thiazol-5-yl)benzonitrile (**3f**) ^{1}H NMR*

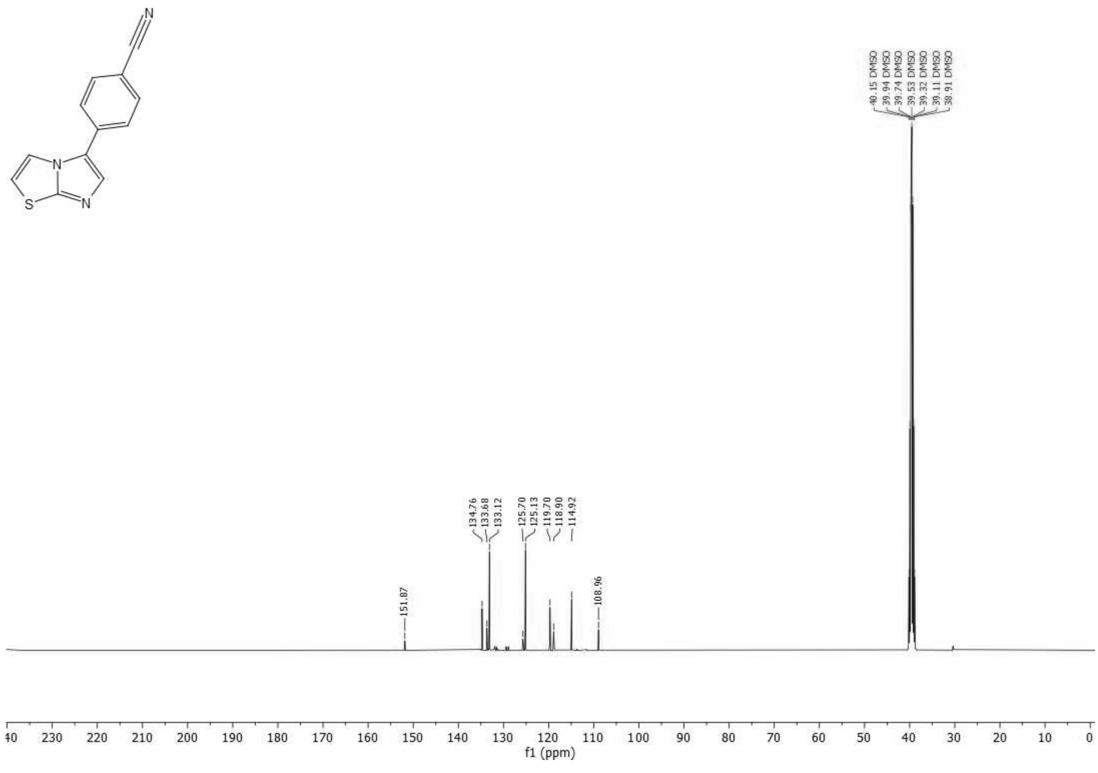

*Figure S103 4-(imidazo[2,1-b]thiazol-5-yl)benzonitrile (**3f**) ^{13}C NMR*

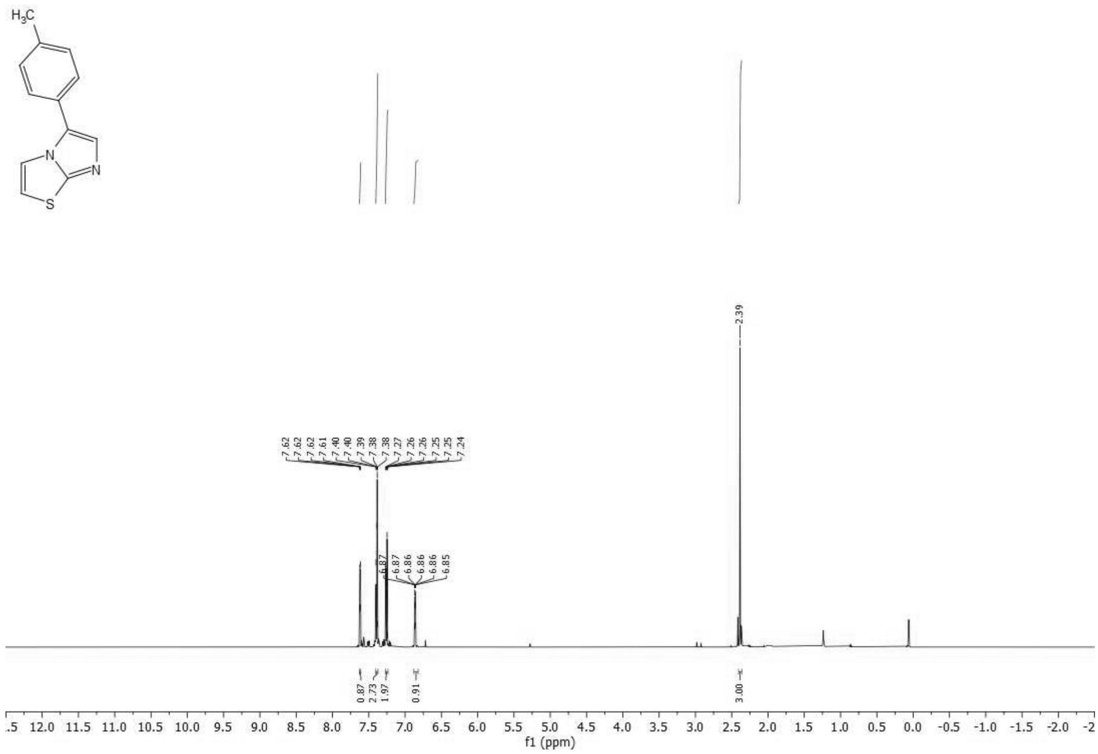

*Figure S14 5-(p-tolyl)imidazo[2,1-b]thiazole (**3g**) ^{1}H NMR*

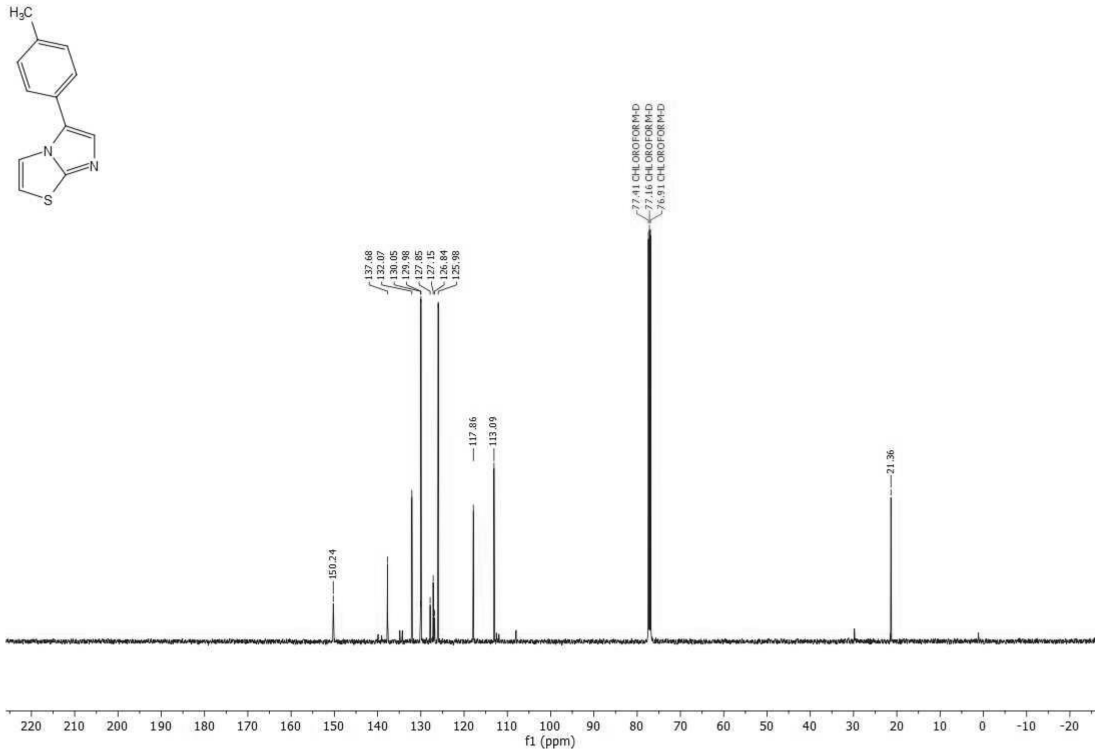

*Figure S15 5-(p-tolyl)imidazo[2,1-b]thiazole (**3g**) ^{13}C NMR*

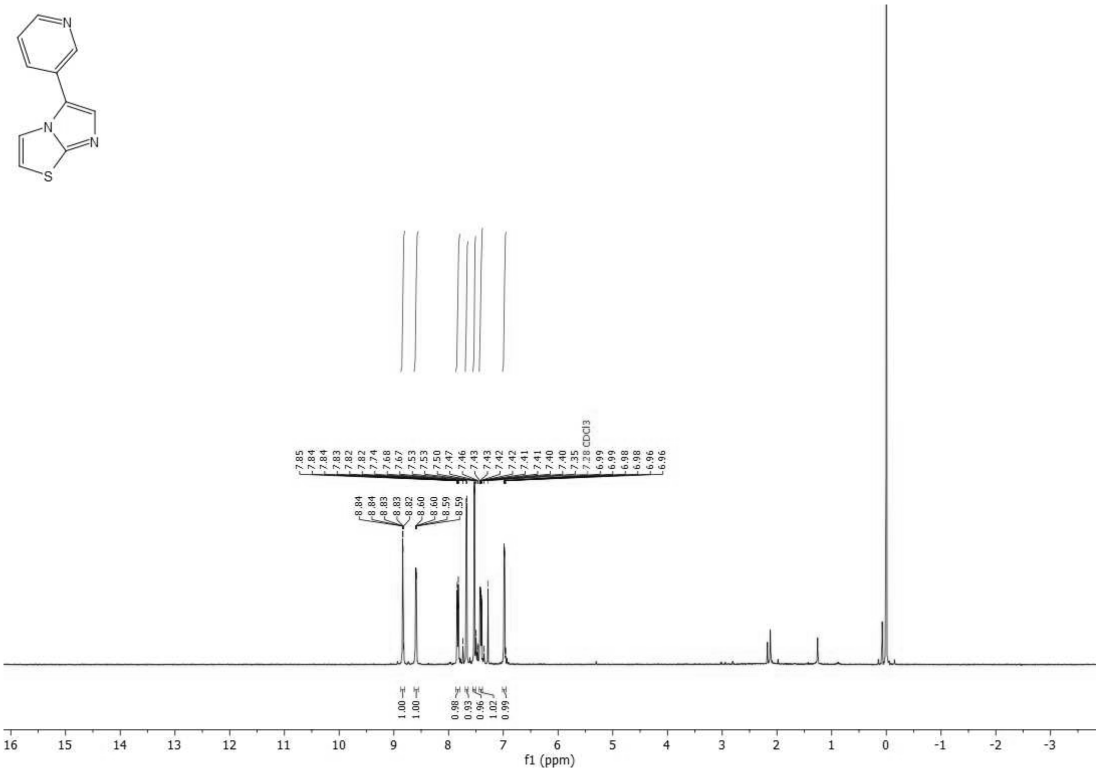

*Figure S16 5-(pyridin-3-yl)imidazo[2,1-b]thiazole (**3h**) ^{1}H NMR*

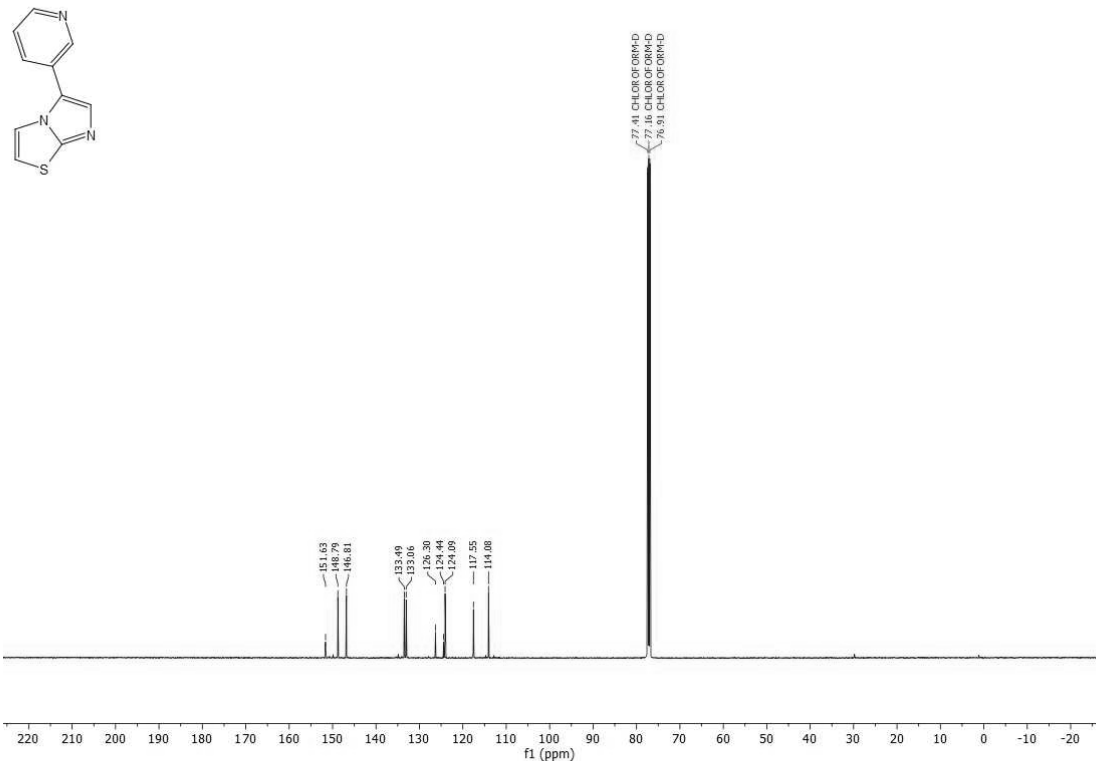

*Figure S17 5-(pyridin-3-yl)imidazo[2,1-b]thiazole (**3h**) ^{13}C NMR*

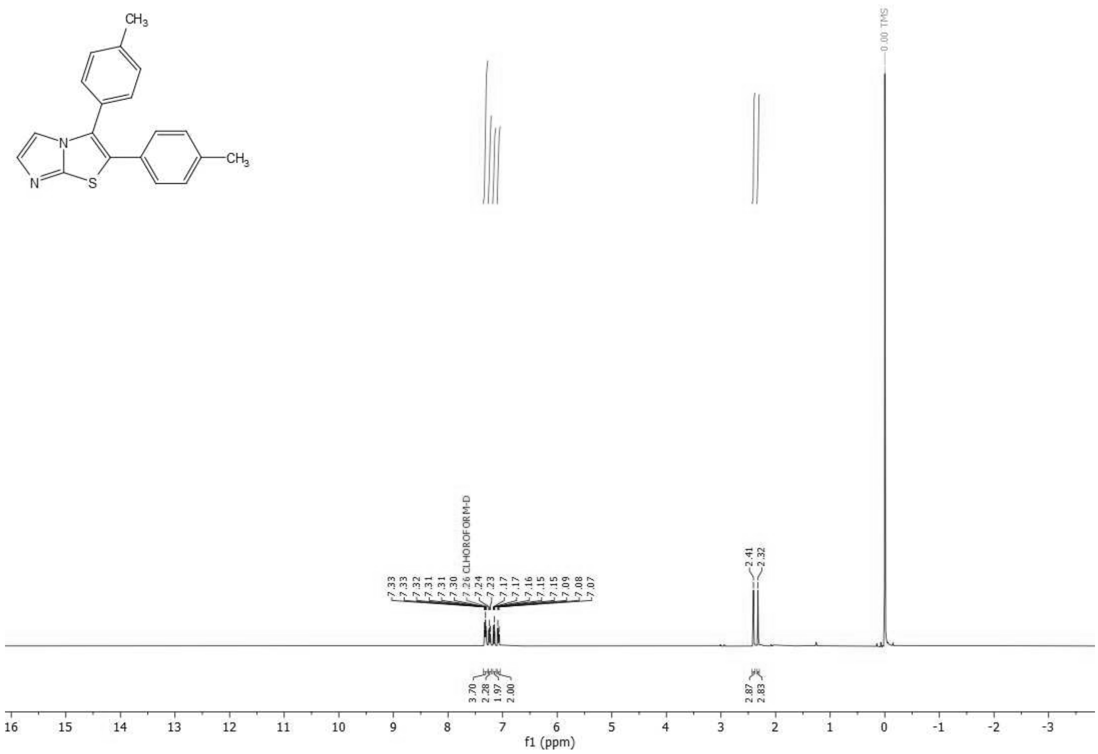

*Figure S18 2,3-bis(4-methylphenyl)imidazo[2,1-b]thiazole (**7a**) ^{1}H NMR*

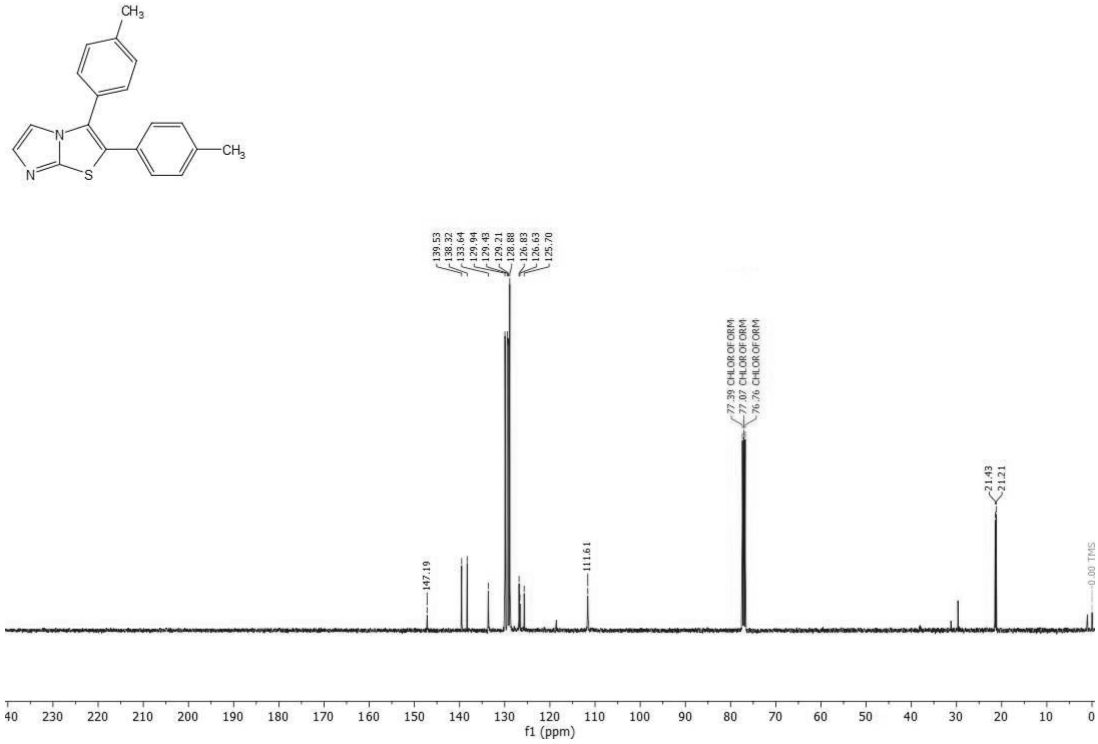

*Figure S19 2,3-bis(4-methylphenyl)imidazo[2,1-b]thiazole (**7a**) ^{13}C NMR*

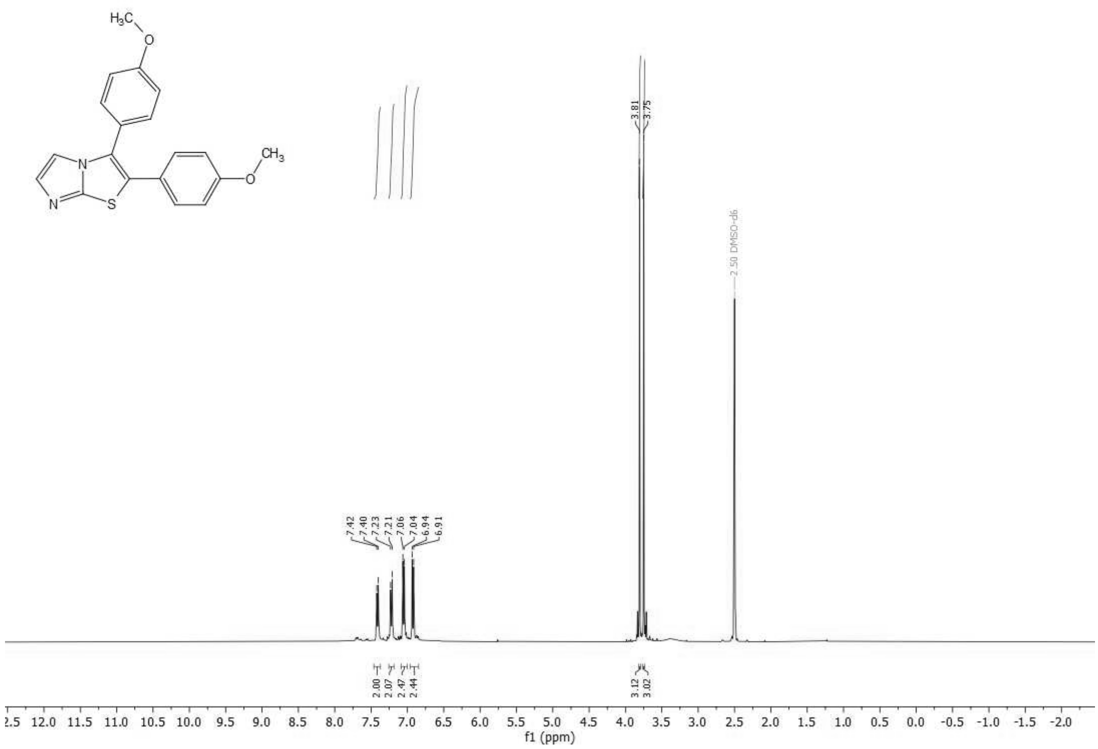

*Figure S20 2,3-bis(4-methoxyphenyl)imidazo[2,1-b]thiazole (**7b**) ^{1}H NMR*

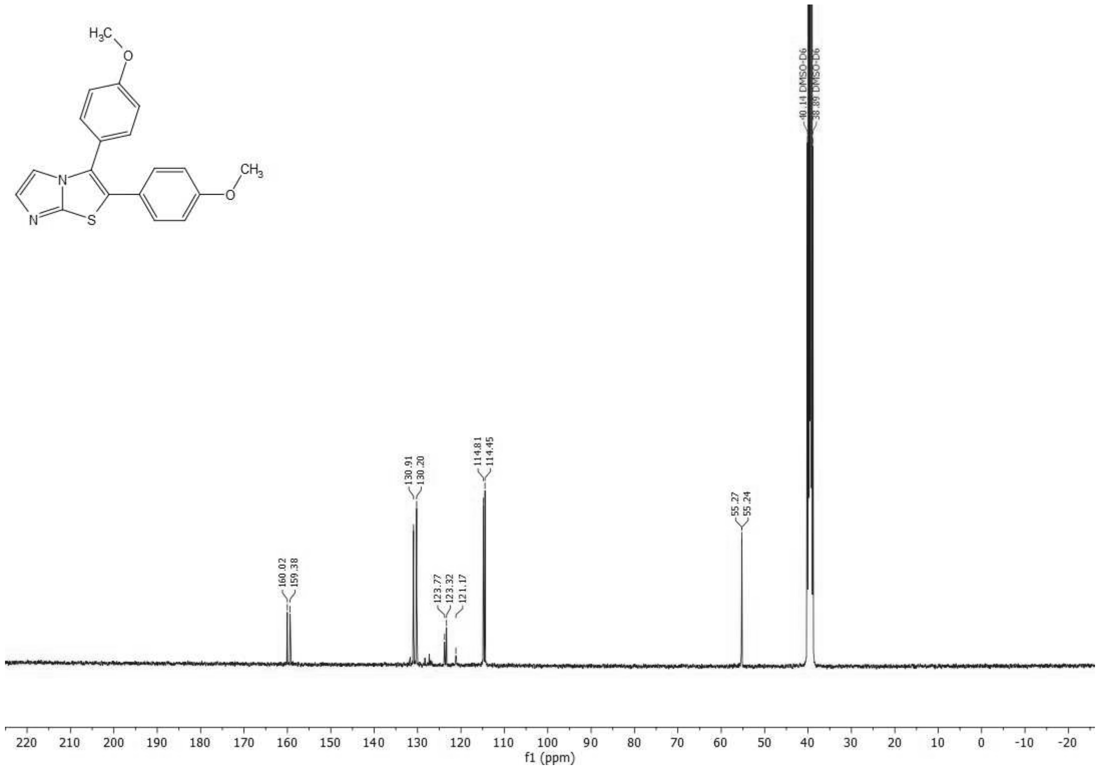

*Figure S21 2,3-bis(4-methoxyphenyl)imidazo[2,1-b]thiazole (**7b**) ^{13}C NMR*

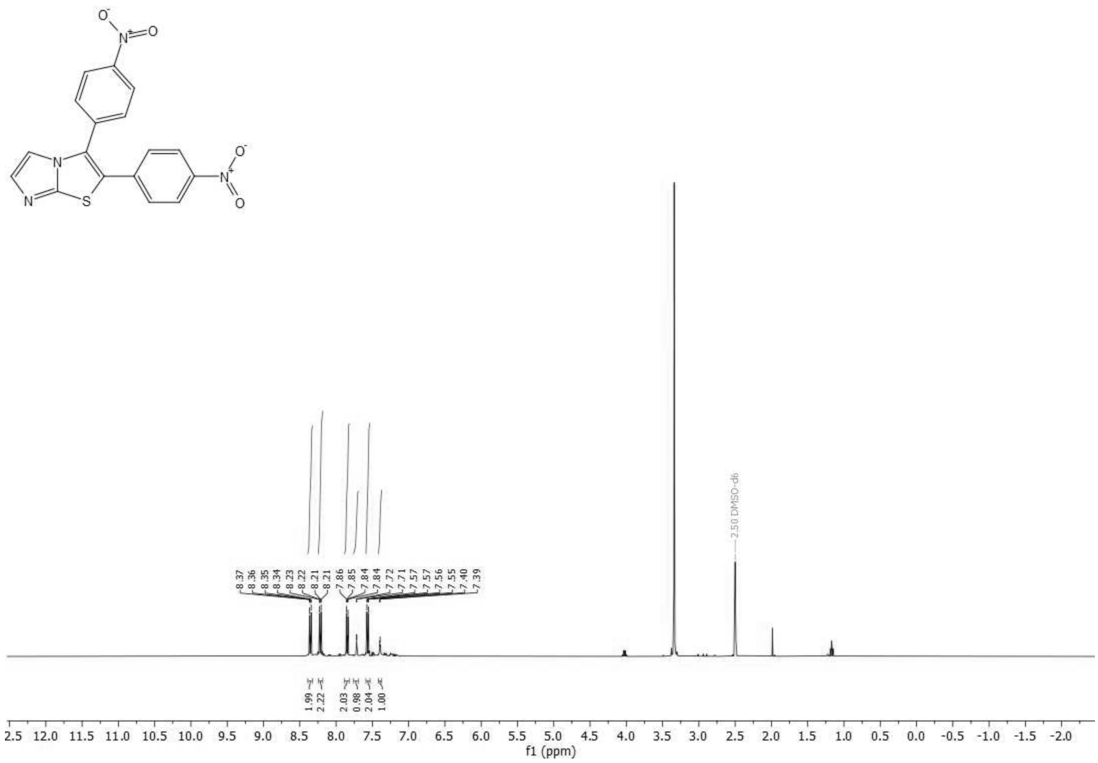

*Figure S22 2,3-bis(4-nitrophenyl)imidazo[2,1-b]thiazole (**7c**) ^{1}H NMR*

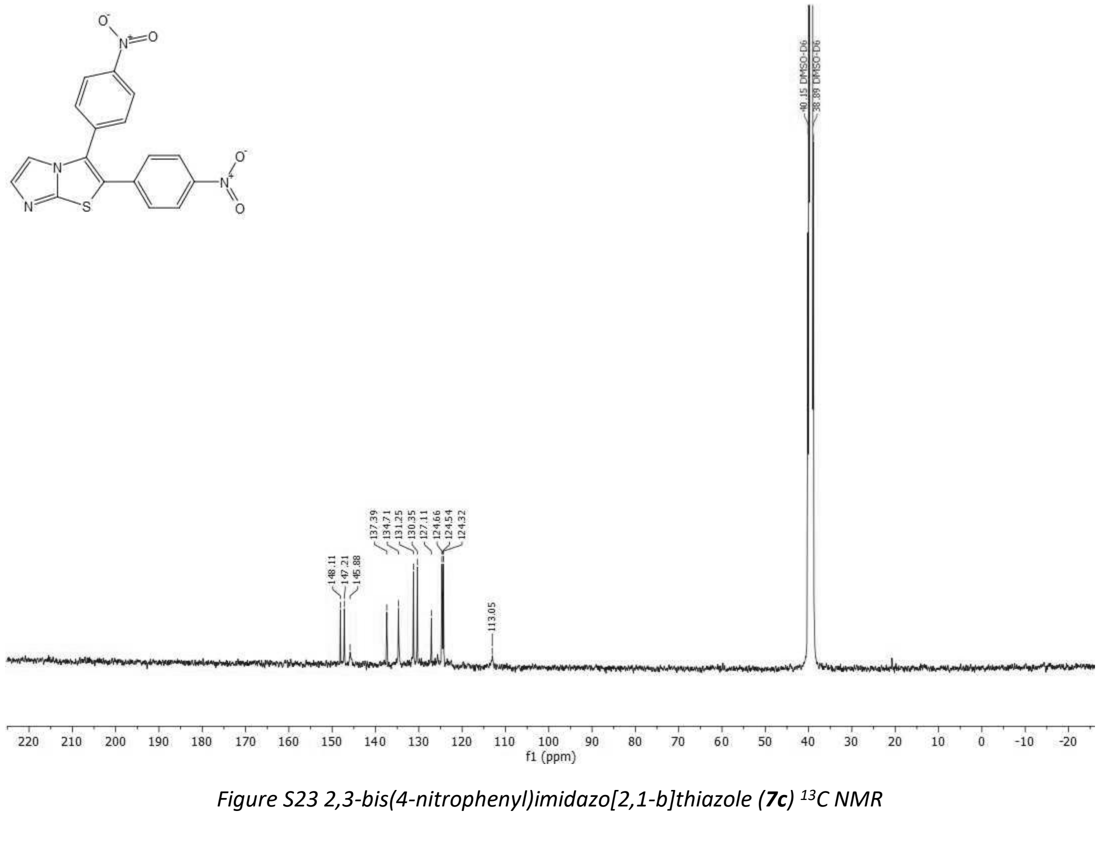

*Figure S23 2,3-bis(4-nitrophenyl)imidazo[2,1-b]thiazole (**7c**) ^{13}C NMR*

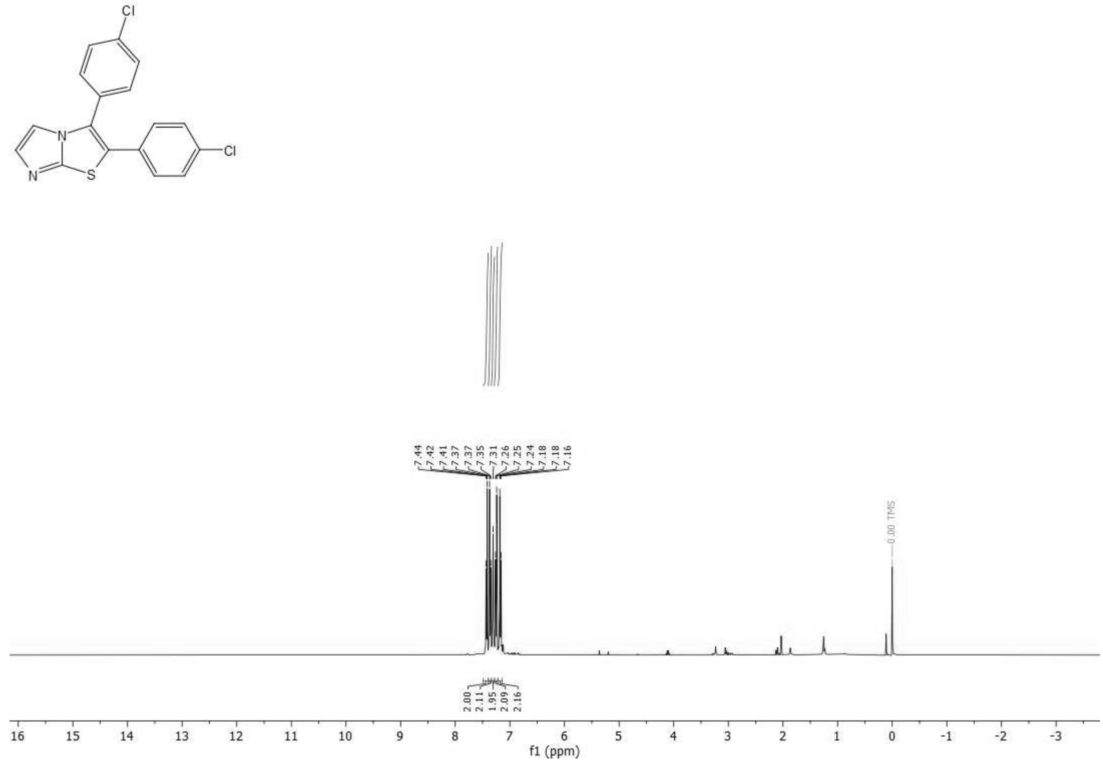

*Figure S24 2,3-bis(4-chlorophenyl)imidazo[2,1-b]thiazole (**7d**) ^{1}H NMR*

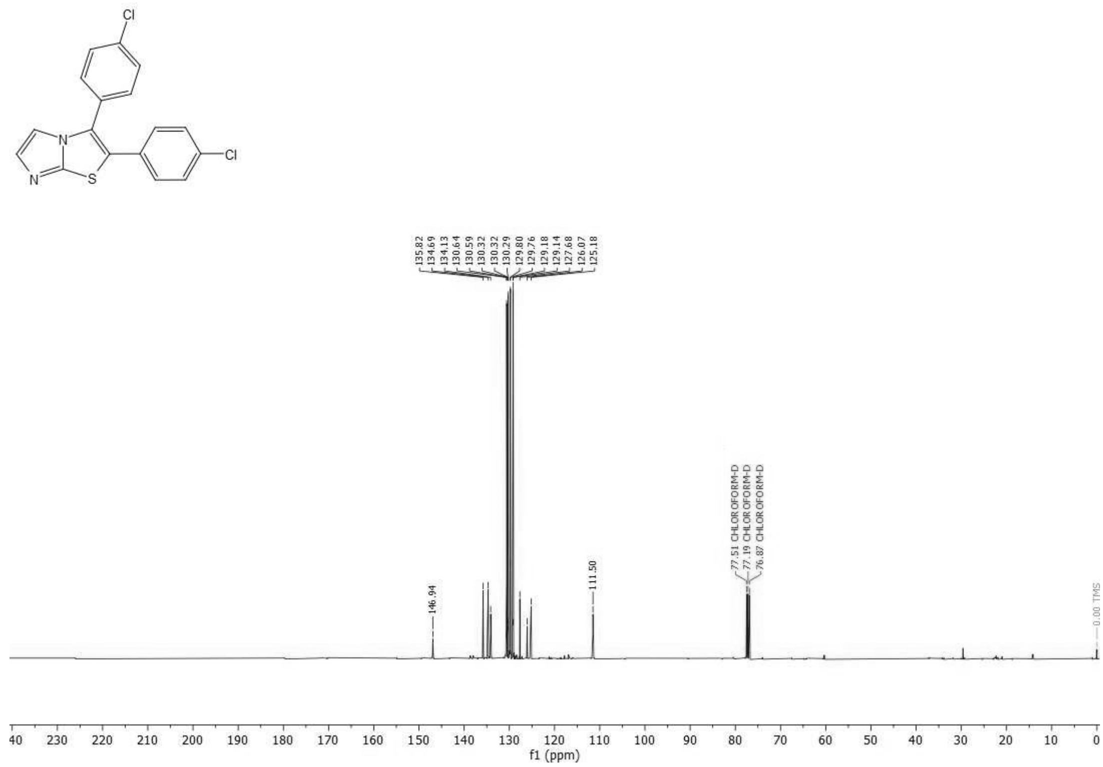

*Figure S25 2,3-bis(4-chlorophenyl)imidazo[2,1-b]thiazole (**7d**) ^{13}C NMR*

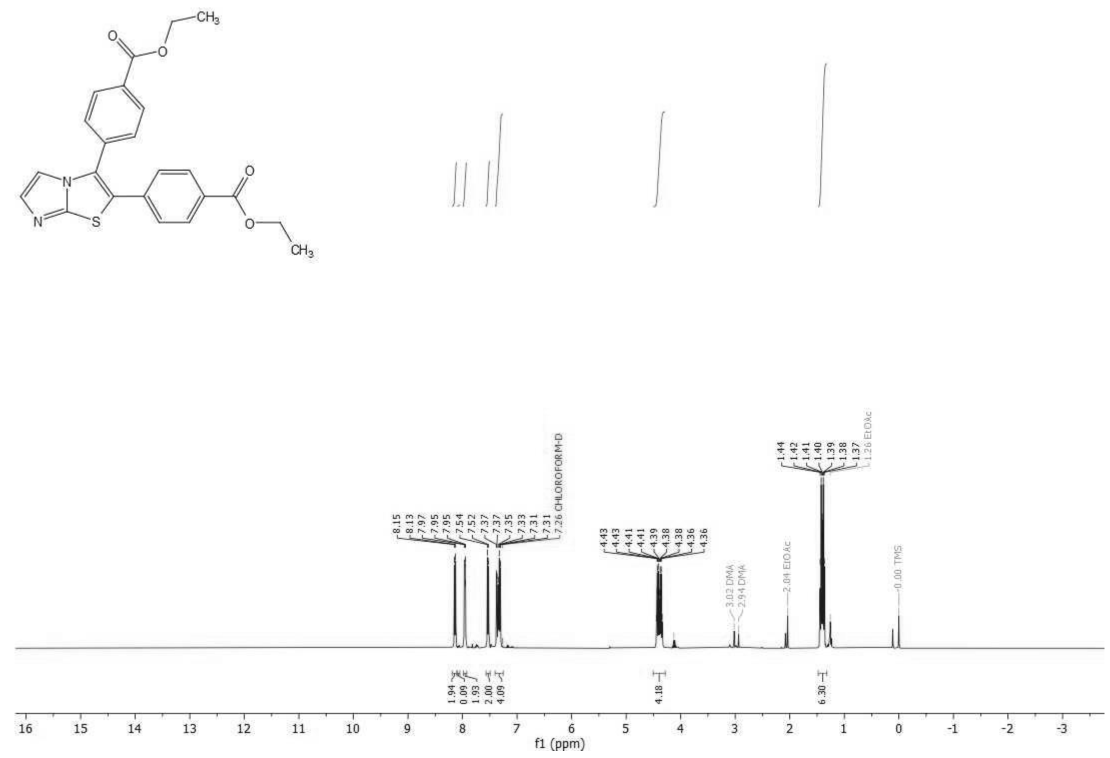

*Figure S26 Diethyl 4,4'-(imidazo[2,1-b]thiazole-2,3)dibenzoate (**7e**) ^{1}H NMR*

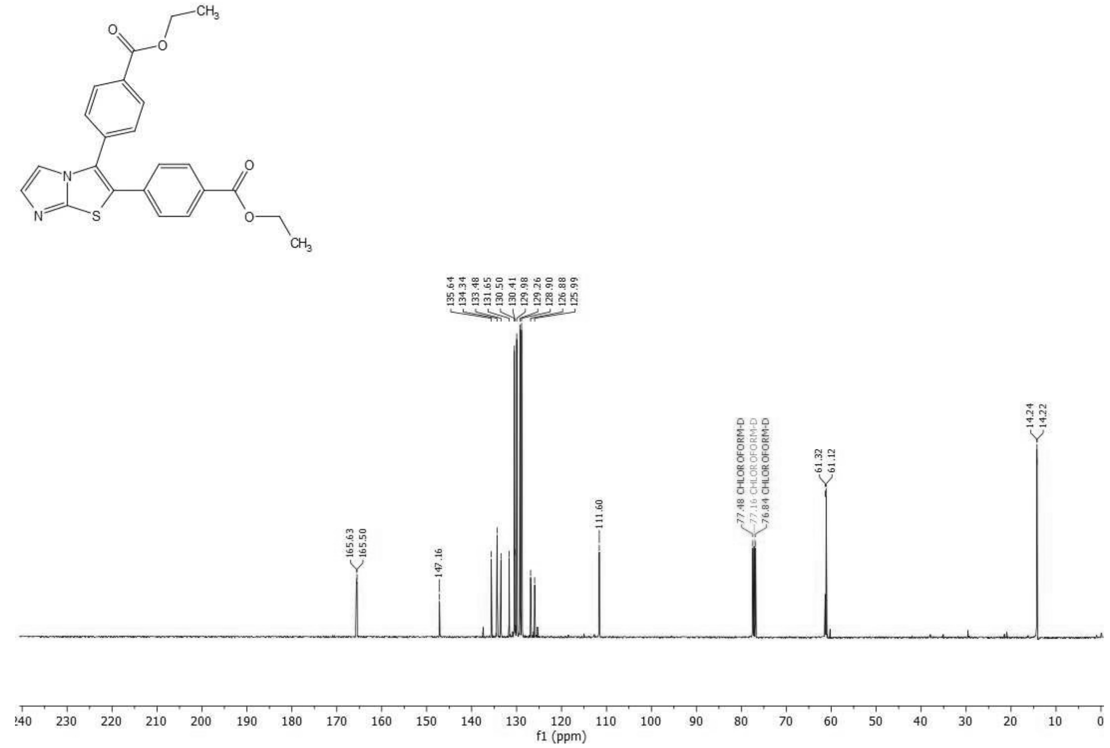

*Figure S27 Diethyl 4,4'-(imidazo[2,1-b]thiazole-2,3)dibenzoate (**7e**) ^{13}C NMR*

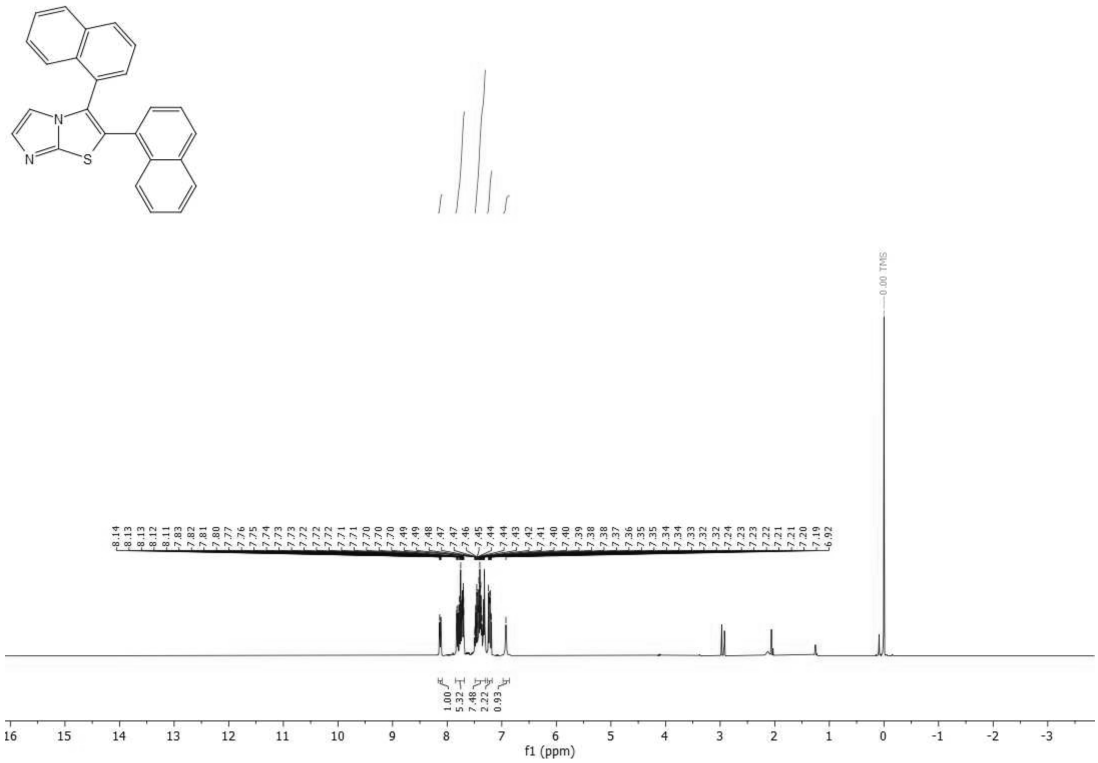

*Figure S11 2,3-bis((1-naphthalen)imidazo[2,1-b]thiazole (**7f**) ^{1}H NMR*

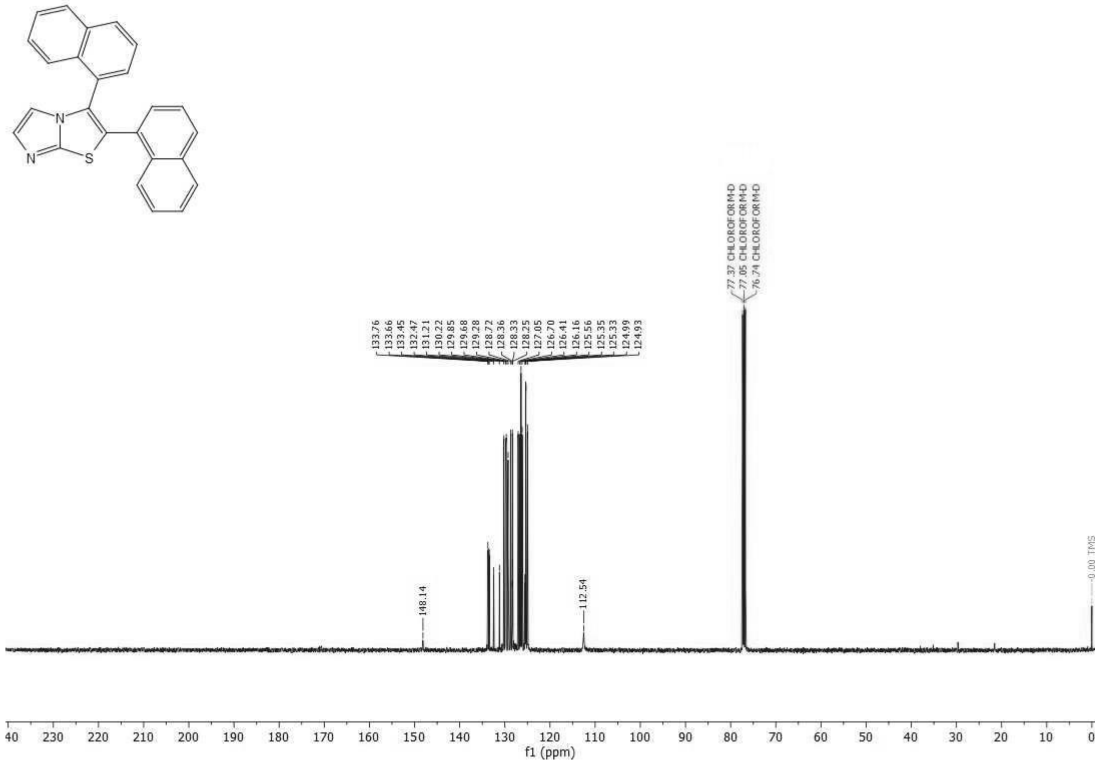

*Figure S12 2,3-bis((1-naphthalen)imidazo[2,1-b]thiazole (**7f**) ^{13}C NMR*

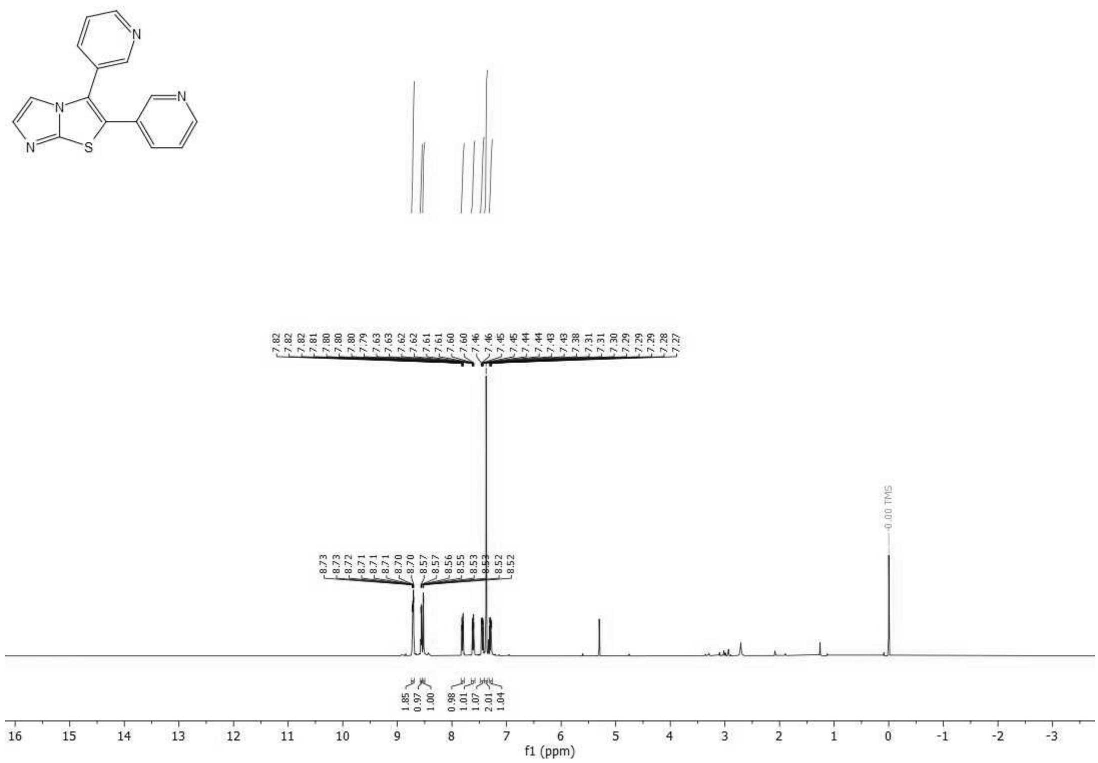

*Figure S30 2,3-di(3-pyridin)imidazo[2,1-b]thiazole (**7g**) ^{1}H NMR*

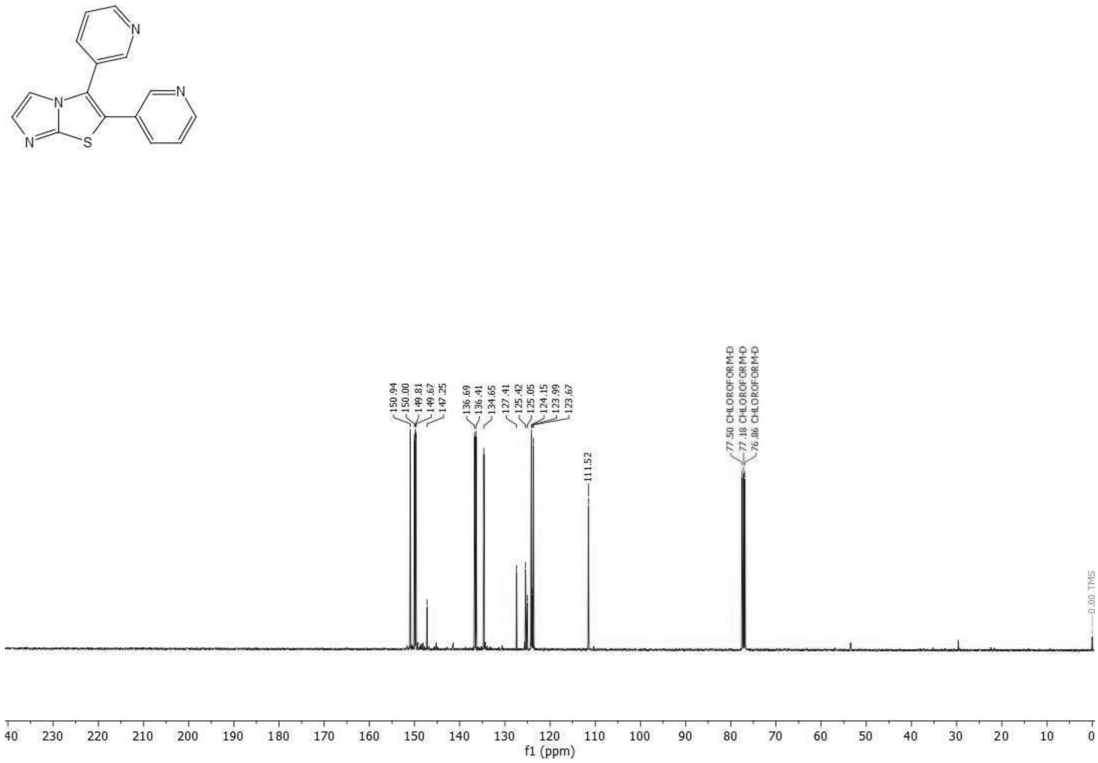

*Figure S31 2,3-di(3-pyridin)imidazo[2,1-b]thiazole (**7g**) ^{13}C NMR*

References

[1] L. Marchetti, L. Pentimalli, P. Lazzaretti, L. Schenetti, F. Taddei, *J. C. S. Perkin* **1973**, 1926-1929.

[2] A. D. Becke, *The Journal of Chemical Physics* **1993**, *98*, 5648-5652.

[3] R. Krishnan, J. S. Binkley, R. Seeger, J. A. Pople, *The Journal of Chemical Physics* **1980**, *72*, 650-654.

[4] J. Tomasi, B. Mennucci, R. Cammi, *Chem Rev* **2005**, *105*, 2999-3093.

[5] A. V. Marenich, C. J. Cramer, D. G. Truhlar, *J Phys Chem B* **2009**, *113*, 6378-6396.

[6] M. A. M. J. Frisch, G. W. Trucks, H. B. Schlegel, G. E. Scuseria, M. Robb, J. R. Cheeseman, G. Scalmani, V. Barone, G. A. Petersson, H. Nakatsuji, X. Li, H. P. Caricato, A. V. Marenich, J. Bloino, B. G. Janesko, R. Gomperts, B. Mennucci, F. Hratchian, J. V. Ortiz, A. F. Izmaylov, J. L. Sonnenberg, D. Williams-Young, F. Ding, V. G. Lipparini, F. Egidi, J. Goings, B. Peng, A. Petrone, T. Henderson, D. Ranasinghe, R. F. Zakrzewski, J. Gao, N. Rega, G. Zheng, W. Liang, M. Hada, M. Ehara, K. Toyota, J. J. Hasegawa, M. Ishida, T. Nakajima, Y. Honda, O. Kitao, H. Nakai, T. Vreven, K. Throssell, K. N. A. Montgomery, Jr., J. E. Peralta, F. Ogliaro, M. J. Bearpark, J. J. Heyd, E. N. Brothers, A. P. Kudin, V. N. Staroverov, T. A. Keith, R. Kobayashi, J. Normand, K. Raghavachari, R. Rendell, J. C. Burant, S. S. Iyengar, J. Tomasi, M. Cossi, J. M. Millam, M. Klene, C. Adamo, D. J. Cammi, J. W. Ochterski, R. L. Martin, K. Morokuma, O. Farkas, J. B. Foresman, Fox, **2016**.
